# Multi-omics analysis identifies TLRscore for prognostic prediction and highlights TLR8 in macrophage-mediated antitumor immunity of lung adenocarcinoma

**DOI:** 10.3389/fimmu.2026.1711401

**Published:** 2026-02-10

**Authors:** Ang Li, Jiawei Liu, Hongjiao Wu, Ye Jin, Hongmei Zhang, Zhi Zhang, Shunli Jiang, Xuemei Zhang

**Affiliations:** 1 School of Public Health, North China University of Science and Technology, Tangshan, China; 2 College of Life Science, North China University of Science and Technology, Tangshan, China; 3 Department of Medical Oncology, Affiliated Tangshan Gongren Hospital, North China University of Science and Technology, Tangshan, China; 4 Department of Chemoradiation, Affiliated Hospital of North China University of Science and Technology, Tangshan, China; 5 School of Clinical Medicine, North China University of Science and Technology, Tangshan, China; 6 Department of Public Health, Jining Medical University, Jining, Shandong, China

**Keywords:** immunotherapy, lung adenocarcinoma, macrophages, TLR8, TLRscore

## Abstract

**Background:**

Toll-like receptors (TLRs) are key mediators of innate and adaptive immunity. Understanding their role in tumor immunity is essential for improving checkpoint blockade therapies.

**Methods:**

Using TCGA data from 32 cancers, we assessed TLR expression, prognosis, and epigenetic changes. A TLRscore was constructed with ssGSEA to evaluate associations with immune infiltration, survival, drug sensitivity, and immunotherapy outcomes, validated in lung adenocarcinoma (LUAD) cohorts. TLR8 polymorphism was genotyped by PCR-RFLP, and the rs3761624 variant was functionally analyzed by luciferase assay. Functional assays in LUAD cells and macrophages treated with the TLR8 agonist Motolimod examined proliferation, migration, invasion, phagocytosis, mitochondrial activity, and ROS generation.

**Results:**

TLRs showed altered expression, frequent mutations, copy number variations, and methylation regulation in cancer. High TLRscore predicted favorable prognosis, increased immune infiltration, and improved immunotherapy response in LUAD. TLR8 was the most immunologically relevant, strongly linked to macrophage infiltration, PD-L1 expression, and T-cell activity. The rs3761624 SNP suppressed TLR8 transcription via enhanced NR1D1 repression. Motolimod reprogrammed macrophages toward an M1 phenotype, boosting cytokine secretion, phagocytosis, and antitumor activity, while inhibiting LUAD cell growth. Mechanistically, TLR8 activation in macrophages was associated with reduced mitochondrial membrane potential, increased ROS production, and the acquisition of an M1-like, antitumor phenotype with enhanced phagocytosis and cytokine secretion.

**Conclusion:**

TLRscore is a novel biomarker for prognosis and immunotherapy response, while TLR8 represents a promising therapeutic target in LUAD, providing mechanistic insight into potential combination strategies.

## 1 Introduction

Malignant tumors have always been one of the most threatening public health problem worldwide, with their incidence and mortality rates continuing to increase globally (1). In recent years, research on antitumor immunotherapy has quickly gained prominence due to its efficacy in addressing different types of cancer (2). For instance, immune checkpoint inhibitors (ICIs) have the potential to considerably extend the overall survival (OS) of cancer patients responsive to ICI treatment (3, 4). However, many patients exhibit minimal or no response to these therapies (5–7). Earlier research has identified various factors related to the responses of cancer patients, including tumor mutational burden (TMB) (8), eosinophilic count (9), immune checkpoint proteins (10–12) and glycolysis activity (13). However, these indicators in predicting effectiveness of immunotherapy still have a significant limitation due to the uncertainty of clinical response. Therefore, many studies have attempted to establish a novel evaluation system of tumor microenvironment and therapeutic response to immunotherapy. It is important to find ways predicting positive responder and further develop new way to boost the effectiveness of cancer immunotherapy.

Toll-like receptors (TLRs) constitute a highly conserved family of pattern recognition receptors that recognize pathogen-associated molecular patterns (PAMPs) and damage-associated molecular patterns (DAMPs), thereby initiating innate immune responses and shaping adaptive immunity (14). Aberrant TLR signaling has been increasingly implicated in carcinogenesis, tumor progression, and modulation of antitumor immunity. Depending on the context, TLRs can exert dual functions: they may promote inflammation-driven tumor growth and immune escape, or alternatively enhance immune activation and tumor suppression (15–17). For instance, activation of TLR4 and TLR9 in tumor cells has been linked to tumor-promoting inflammation and resistance to apoptosis, whereas TLR3 and TLR7/8 activation in myeloid cells can trigger type I interferon production and potent antitumor immune responses (18–20). These observations highlight the complexity and context-dependency of TLR signaling in the tumor microenvironment.

Among the TLRs, TLR8 is of particular interest due to its selective expression pattern and immunomodulatory potential. TLR8 expression is largely restricted to monocytes, macrophages, and myeloid dendritic cells, where its activation elicits pro-inflammatory cytokine release and promotes antigen-presenting functions (20, 21). Recent studies have demonstrated that pharmacologic activation of TLR8 can enhance antigen presentation, and augment natural killer (NK) cell–mediated antibody-dependent cellular cytotoxicity (22, 23). However, the mechanisms by which TLR8 activation reshapes macrophage function in the tumor context, particularly in lung adenocarcinoma (LUAD), remain poorly characterized.

Recent advances in cancer immunology have underscored the central role of macrophage polarization and metabolism in dictating tumor outcomes. Tumor-associated macrophages (TAMs) often adopt an M2-like, immunosuppressive phenotype that promotes tumor progression and limits responses to immune checkpoint blockade (24). In contrast, M1-like macrophages exert robust antitumor functions through secretion of pro-inflammatory cytokines, phagocytic activity, and stimulation of cytotoxic lymphocytes (25). Accumulating evidence suggests that mitochondrial integrity and moderate reactive oxygen species (ROS) production are indispensable for sustaining the M1 phenotype and effective antitumor immunity (22, 26). Yet, whether TLR8 agonists can harness this mitochondrial-ROS axis to reprogram macrophages toward an M1 state has not been systematically addressed.

Lung adenocarcinoma, the most common subtype of non-small cell lung cancer, remains a leading cause of cancer-related death despite modern therapies. Tumor-intrinsic pathways such as apoptosis and oncogenic signaling are key determinants of treatment resistance: Zheng et al. identified a GSK3β/ITCH/c-FLIP axis that counteracts TRAIL-induced apoptosis in LUAD cells (27), and Cai et al. showed that the irreversible EGFR inhibitor CL-387785 suppresses growth, invasion and enhances radiosensitivity in EGFR-mutant cells (28). However, compared with these tumor-cell–focused mechanisms, much less is known about how TLRs, key innate immune sensors, shape the LUAD immune microenvironment and influence prognosis and response to immunotherapy. In this study, we sought to fill this critical knowledge gap by performing a comprehensive pan-cancer analysis of TLR expression, mutation patterns, and prognostic associations, and by developing a TLR-associated activity score (TLRscore) to assess their clinical relevance. Finally, we focused on the role of TLR8 in LUAD, evaluating its impact on the tumor immune microenvironment. Our results provide mechanistic insights into how TLR8-driven macrophage reprogramming contributes to antitumor immunity.

## 2 Materials and methods

### 2.1 Datasets and source

The expression data of mRNA, alterations in copy number, 450K DNA methylation data, mutation records, and clinical information related to 32 different solid tumor (exclude blood tumor LAML) sourced from the Cancer Genome Atlas (TCGA) database (https://portal.gdc.cancer.gov/). Microarray datasets including gene expression profiles and corresponding clinical information data of 5 different LUAD cohorts (GSE72094, GSE13213, GSE14814, GSE11969 and GSE68465) downloaded from the GEO database were also involved in our study. Additionally, our research investigated three immunotherapy cohorts (GSE78220, GSE91061 and IMvigor210). Among them, IMvigor210 is obtained from the R packaging “IMvigor210 CoreBiologies”. The single cell was analyzed using the TISCH2 database (http://tisch.comp-genomics.org/home/). Data related to the immune system, such as the proportions of different immune cell types and the immunophenoscore, were sourced from TCIA (https://tcia.at/home).

### 2.2 Data establishing the toll-like receptor score model

We constructed an expression-based TLRscore using the single-sample gene set variation analysis (ssGSEA) algorithm implemented in the “GSVA” R package, applied to the gene set comprising TLR1-TLR10. For each sample, ssGSEA estimated an enrichment score reflecting the coordinated expression of the 10 TLR genes; this ssGSEA derived value was used as the primary TLRscore throughout the study. Thus, TLRscore represents a relative, composite estimate of overall TLR abundance rather than a direct measurement of receptor activation. To evaluate the robustness of TLRscore to the choice of scoring algorithm, we additionally derived TLR signatures using z-score–based average (mean of standardized expression values of TLR1-TLR10) on the same TLR1-TLR10 gene set. We compared their concordance with the ssGSEA-based TLRscore using Spearman correlations.

### 2.3 Analysis of somatic copy-number alterations and single nucleotide variations

The amplification and deletion of both heterozygosity and homozygosity were considered to enhance the SCNA for each gene, with more than five percent deemed as high-frequency SCNA. To evaluate the connection between SCNA and gene expression, we calculated Pearson’s correlation coefficient by utilizing the expression values in conjunction with the corresponding copy number segment values for gene. Additionally, SNV data were gathered from 32 different cancer types available in the TCGA database. An oncoplot depicting SNVs was created utilizing the maftools package.

### 2.4 DNA methylation analysis

The R package named “IlluminaHumanMethylation-450kanno.ilmn12.hg19” was utilized, which is available through Bioconductor, was utilized for the purpose of annotating the methylation probes associated with the promoter regions of each gene. To evaluate the varying methylation patterns of these genes in tumor versus normal samples, the Wilcoxon signed-rank test was utilized. This rigorous statistical approach allowed for the identification of genes that exhibited significant hypomethylation or hypermethylation. Furthermore, a Pearson’s correlation analysis was performed to investigate the relationship between the transcriptional expression levels of TLRs and the Beta values indicative of promoter DNA methylation.

### 2.5 Analysis of the tumor mutation status in different TLRscore group

To identify significantly mutated genes, the data regarding somatic mutations (nonsynonymous mutations and synonymous mutations) collected through TCGA, and analysis was conducted comparing the different TLRscore groups. Additionally, the interaction effects of gene mutations were examined through the use of the maftools package. For the purposes of this analysis, only genes that had undergone mutations more than 30 times in at least one of the two groups were included.

### 2.6 Prognostic analysis of the TLRscore

The analyses of univariate cox regression and Kaplan-Meier were conducted using the R packages “survminer” and “survival” to investigate the association between TLRscore and patient survival, which encompasses OS and disease-specific survival (DSS).

### 2.7 Construction of nomogram

A nomogram is a prognostic prediction tool constructed based on a multivariate Cox proportional hazards regression model. The model integrates three independent prognostic factors (age, tumor stage, and TLRscore), every factor was associated with a specific score, which contributed to the cumulative score. The total points were then utilized to predict the 1-year, 3-year, and 5-year OS rates; a higher score correlated with a decreased survival rate.

### 2.8 Gene set variation analysis

The specimens from each type of tumor were categorized into two categories based on the TLRscore, comprising the highest 30% and the lowest 30%. In order to minimize overlaps and redundancies in pathways, each gene set linked to a specific pathway was refined to include only unique genes, with all genes that were associated with two or more pathways being excluded. Subsequently, GSVA was conducted utilizing the R package “GSVA” to investigate the functions related to TLRs. The pathways identified as Hallmark and used in GSVA were sourced from the MsigDB database.

### 2.9 Quantify the immunotherapy response predictor

The Tumor Immune Dysfunction and Exclusion (TIDE) algorithm platform (http://tide.dfci.harvard.edu/) was employed to model intratumoral immune dysfunction and exclusion. Its composite score (TIDEscore) was used to predict patient responses to immune checkpoint inhibitor therapy. Patients with higher TIDEscore values typically exhibit poorer responses to immune checkpoint inhibitors, whereas those with lower scores are more likely to benefit from immunotherapy (29). To forecast how patients might respond to Immune-Checkpoint Blocker (ICB) therapy, we analyzed mRNA-seq data from TCGA-LUAD, GSE72094, and GSE13213 employing the TIDE algorithms. A high TIDE score indicated a higher probability of being an immunotherapy non-responder, whereas a low TIDE score suggested the contrary.

The Immunophenoscore (IPS) (30), a key molecular marker indicative of an enhanced immune response, was employed to analyze the immune landscapes within tumors and the cancer antigenomes. This scoring system was developed based on a collection of genes associated with immunity, divided into four distinct clusters: molecules related to the major histocompatibility complex (MHC), immune checkpoint proteins (CP), effector cells (EC), and suppressor cells (SC). A sample-wise Z score was derived and computed from the LUAD expression data for each group. Subsequently, the weighted average Z score was determined by computing the average of Z scores in each category, resulting in four individual values, along with the cumulative weighted average Z score across the four groups.

### 2.10 Tumor microenvironment characteristics

The algorithms CIBERSORT, ESTIMATE, MCPcounter, ssGSEA, and TIMER were evaluated to analyze cellular components and immune responses in groups with high and low TLR scores, determined by TLR expression levels. A Heatmap was utilized to reveal the differences in immune responses across the various algorithms.

### 2.11 Drug sensitivity analysis

The Genomics of Drug Sensitivity in Cancer (GDSC) database comprises a vast collection of genomic datasets and drug sensitivity information (31). We utilized the “pRRopheticPredict” R package (version 0.5) to construct statistical models leveraging gene expression data derived from a large array of cancer cell lines. Subsequently, we applied these models to the gene expression data of the target samples (32) to assess the variations in IC50 values of 198 drugs between the different TLRscore groups. The selection of the working concentration of glycogen synthase kinase-3 (GSK-3) inhibitor (SB216763) is based on previous publications indicating that 5μM can affect the viability of LUAD cells (33).

### 2.12 Study population

This study includes 400 LUAD cancer patients and 600 healthy controls and the basic characteristics are shown in **Supplementary Table 1**. Cases were collected from Gongren Hospital of North China University of Science and Technology (NCST) in China with histopathological confirmation. Cancer-free healthy controls were collected from Tangshan area. All participants were genetically unrelated Han Chinese. Informed consent was provided by every individual. Participant provides 2 ml venous blood sample for DNA extraction. This research was supported by the Institutional Review Board of North China University of Science and Technology (Ethics Number: 2022027).

### 2.13 The selection of genetic variants

This study utilizes data from the Ensembl and NCBI databases, employing GRCh38 as the reference genome to extract information on single nucleotide polymorphisms (SNPs) of the TLR8 gene within East Asian populations. During the data screening process, the minor allele frequency (MAF) was required to be no less than 0.05, and the polymorphic sites needed to be situated within the promoter region of TLR8. Following a comprehensive literature review, primer design, and analysis of enzyme cleavage sites, three SNPs-rs3764880, rs3761624, and rs5741883-were ultimately selected for further investigation. We employed network-based transcription factor prediction tools, specifically Alibaba 2.1 and JASPAR, to predict the binding sites of transcription factors.

### 2.14 Determination of genotypes

In this research, DNA extracted from the peripheral blood of patients diagnosed with lung adenocarcinoma, as well as from healthy individuals, was analyzed to identify germline polymorphisms associated with the risk of lung adenocarcinoma. It is important to emphasize that the DNA utilized for genotyping was sourced from peripheral blood rather than tumor tissue, thereby primarily reflecting the genetic profile of the lineage, as opposed to somatic variations that may be present in tumor cells. The genotypes of the TLR8 polymorphisms rs3764880, rs3761624, and rs5741883 were determined using polymerase chain reaction (PCR) in conjunction with restriction fragment length polymorphism (RFLP) analysis. Primers were obtained from SinoGenoMax (Beijing, China), with the pairs used being rs3764880F/rs3764880R (5′-GCT ACG TTC TGC TGA TGG TAA A-3′/5′-AGG ATA TTG TCT CTG GTA GGG C-3′), rs3761624F/rs3761624R (5′-AAG CCA TTC TTT GAC TGC TGA C-3′/5′-CGG CCC AGA AGT GAA AAC ATT T-3′), and rs5741883F/rs5741883R (5′-CTC ACG AAT GCT CAG CCA T-3′/5′-AAT CAG ACA CAC ACG ACG T-3′). The PCR was performed in a reaction mixture of 6 μl, containing 20ng of genomic DNA, 0.1μM of each primer, and Taq PCR StarMix (GenStar, China). The PCR cycle included an initial denaturation at 94°C for 5 minutes, followed by 30 cycles of denaturation at 94°C for 20 seconds, annealing at 59°C for 30 seconds, and extension at 72°C for 35 seconds, concluding with a final extension at 72°C for 5 minutes. The PCR products corresponding to rs3764880, rs3761624, and rs5741883 of the amplified TLR8 gene were subjected to digestion with NIA III and NSP I (NEB, Ipswich, USA), and the resultant fragments were evaluated through agarose gel electrophoresis. To ensure the accuracy and reliability of the genotyping results obtained from PCR-RFLP, a rigorous validation procedure was implemented. We randomly selected 10% of the samples from each identified genotype for retesting to assess the consistency and reproducibility of our findings.

### 2.15 Luciferase reporter assay

For the TLR8 promoter activity analysis, we created reporter constructs that contained a 1367 bp fragment from the promoter region of TLR8.The primers used were TLR8-PF (5′-CGGGGTACCCTGCACTCGATCCATTCTTACTTA-3′) and TLR8-PR (5′-CCGCTCGAGGGGAACTTGTGATTTCTTAGCTGT-3′), as shown by the underlined sequences demonstrated that these constructs incorporated cloning sites for *Kpn I* and *Xho I* (NEB, Ipswich, USA). Subsequently, the PCR product was integrated into the pGL3-basic reporter vector (Promega, Madison, USA). We designed this construct as pGL3-rs3761624A based on the sequence results. Subsequently, we obtained pGL3-rs3761624G plasmids by site-specific mutagenesis using the pGL3-rs3761624 A vector as template. All the constructed vectors were verified via DNA sequencing. A549 cell lines (3×10^5^) were plated in 24 well plates. Once the cell confluence reached approximately 80%, transfection was carried out. Specifically, pGL3-TLR8pro and pRL-SV40 plasmid were transfected into the cells using the Lipofectamine 2000 reagent (Thermo Fisher Scientific, USA). After a 24 hour incubation period, the cells were collected, and the fluorescence signal was detected via a dual luciferase reporter assay (Promega, USA).

### 2.16 Cell culture

A549, NCI-H1299 and THP-1 cells were purchased from Procell (Hubei, China). The cells were grown in RPMI-1640 medium that contained 10% fetal bovine serum (Tianhang Biotechnology, Zhejiang, CHN) and antibiotics, in a humidified incubator maintained at 37°C with 5% CO_2_. The selective TLR8 agonist Motolimod (VTX-2337) was used to activate TLR8 signaling in macrophages. The working concentration was chosen based on previously publication showing that Motolimod in the low micromolar range effectively triggers TLR8-dependent cytokine production in human immune cells without inducing nonspecific cytotoxicity (1–3 μM) (34) Therefore, a concentration of 3 μM was used as the standard dose for all functional assays unless otherwise specified.

### 2.17 Cell proliferation

In order to conduct cell proliferation assays, 5×10³ cells were placed in each well of a 96-well plate and subsequently incubated with varying concentrations of Motolimod in a 37 °C incubator with 5% CO2. The Cell Counting Kit-8 (Mei5 Biotechnology, Beijing, CHN) was utilized to assess cell proliferation at 24, 48, and 72 hours following the guidelines provided by the manufacturer.

### 2.18 Cell migration and invasion assays

For cell migration assays, cells (1.5×10^5^ cells/200µl) resuspended in serum-free Ham’s F12K medium were plated in the upper chamber without Matrigel (Corning, NY, USA). For cell invasion assays, cells (1.5×10^5^ cells/200µl) were resuspended in serum-free Ham’s F12K medium and seeded in the upper chamber coated with Matrigel. Medium containing 10% FBS (600µL) was added to the lower chamber. Following 24 hours of incubation at 37°C with 5% CO_2_, the cells were fixed at room temperature with 4% paraformaldehyde for 20 minutes, stained for 15 minutes with 0.5% crystal violet dye, and microscopy images were captured.

### 2.19 Quantitative real-time PCR

RNA from the cells was isolated with TRIzol reagent (Invitrogen, CA, USA) and subsequently reverse-transcribed into cDNA using the RevertAid first-strand cDNA synthesis kit (Thermo Fisher Scientific, MA, USA). After this step, use SYBRGreen PCR Master Mix (Vazyme, China) for real-time quantitative PCR. The reverse transcription and qRT-PCR reaction setups were arranged following the guidelines provided by the manufacturer. To determine the relative levels of gene expression, the 2^-△△ct^ method was employed, with normalization to GAPDH expression levels for every sample.

### 2.20 Mitochondrial membrane potential detection

In a 6-well plate, inoculate macrophages that have been treated either with Motolimod or left untreated. Following this, incubate the cells with the JC-1 fluorescent probe (Solarbio, Beijing, China) in a 5% CO_2_ environment at 37°C for 15 minutes, as per the manufacturer’s guidelines. After incubation, rinse the cells twice with washing buffer and then proceed to observe them. In mitochondria that have a high membrane potential, JC-1 appears as aggregates emitting red fluorescence, whereas in mitochondria with a low membrane potential, it exists as monomers that emit green fluorescence.

### 2.21 Reactive oxygen species level detection

Seed M0 macrophages in a 6-well plate, applying either Motolimod treatment or leaving them untreated, and then incubate in serum-free medium at 37°C with 10µM of the fluorescent probe 2’, 7’-dichlorofluorescein diacetate (Invitrogen, MA, USA) protected from light for 30 minutes. Following the incubation period, rinse the cells three times with phosphate-buffered saline (PBS) to eliminate any unbound probes, and promptly assess ROS-dependent DCF fluorescence (FITC channel, excitation at 488nm, emission at 525nm) using a fluorescence microscope.

### 2.22 Detection of macrophage phagocytic ability

Macrophages should be inoculated in a 6-well plate at a density of 1.5×10^5^ cells per well. Next, those macrophages, either treated with Motolimod or left untreated, are to be incubated in fresh medium that contains the lipophilic fluorescent dye DiI (Invitrogen, USA) at a temperature of 37°C in an atmosphere with 5%CO_2_ for a duration of 30 minutes. Following the incubation period, the cells must be washed three times with preheated PBS to eliminate any unbound dyes. After this, co-culture the macrophages with lung adenocarcinoma cells that have been stained with DAPI (Invitrogen, USA) for nuclear visualization. Lastly, cell images should be captured under a fluorescence microscope to observe DAPI (blue for nuclear staining), Dil (red to indicate dye uptake), and merged fields.

### 2.23 Statistical analysis

Statistical evaluations and graphical representations were conducted using GraphPad Prism 8.0 (GraphPad Software, CA, USA) and R version 3.6.1. Pearson correlation analysis was employed to assess the correlation. The comparisons between two groups were assessed for statistical significance through Student’s t-test. For comparisons among more than two independent groups, non-parametric data were analyzed using the Kruskal–Wallis test. When this test was significant, Dunn’s *post hoc* test was applied for pairwise group comparisons, with Bonferroni correction to adjust for multiple testing. For estimating overall survival, the Kaplan-Meier method was used alongside Cox regression analysis. A *P*-value of less than 0.05 was deemed significant.

## 3 Results

### 3.1 Aberrant expression and genetic alterations of TLRs across cancer types

To obtain an overview of TLR dysregulation in human cancer, we analyzed the expression of 10 TLR genes across 32 TCGA tumor types. Each TLR was differentially expressed in at least one type of cancer, and certain TLRs revealed similar expression patterns. In particular, TLR2, TLR7 and TLR8 were significantly upregulated in 14, 11 and 10 types of cancers, respectively, whereas TLR3 and TLR4 were downregulated in 16 and 13 cancers (**Supplementary Figure S1A**). Moreover, various TLRs exhibited distinct patterns specific to different cancer types that have not been thoroughly characterized before. For example, most TLRs showed significant upregulation in Glioblastoma multiforme (GBM) and Kidney renal clear cell carcinoma (KIRC), but was downregulated in Colon adenocarcinoma (COAD), Lung squamous cell carcinoma (LUSC), Pancreatic adenocarcinoma (PAAD) and Rectum adenocarcinoma (READ). Survival analyses further revealed that high expression of TLRs was associated with improved prognosis in Sarcoma (SARC), Skin Cutaneous Melanoma (SKCM), and LUAD, but with poorer outcome in Lower Grade Glioma (LGG), Testicular Germ Cell Tumors (TGCT), and Stomach adenocarcinoma (STAD) (**Supplementary Figure S1B**). Thus, TLR expression displays both cancer-type-specific and context-dependent prognostic effects.

Gene expression is greatly affected by several genetic and epigenetic factors, such as mutation, DNA methylation and copy number variation. In our analysis of methylation, we noted that Toll-like receptors exhibited intricate methylation patterns across 23 different types of cancer (**Supplementary Figure S1C**). For example, TLR3 and TLR9 showed hypermethylation in 23 cancers, but only observed a negative correlation was noted solely between the expression of TLR3 and DNA methylation levels (**Supplementary Figure S1D**). Given that SCNA is crucial for gene expression in tumors, we assessed how SCNA influences the gene expression of TLRs and discovered that TLR3 expression is markedly positively correlated with SCNA in a majority of cancers (**Supplementary Figure S1E**), which indicated that the aberrance SCNA of TLR3 might contribute to the progression of various cancers. Copy number analysis visualized the frequency of somatic copy-number gains and losses affecting each TLR gene across various tumor types. somatic copy-number alterations (>5% of samples) occurred in most tumor types (**Supplementary Figure S1F**), highlighting that SCNAs of TLR genes are common events across multiple tumor types. With the exception of TLR5, TLRs were more prone to copy-number loss than gain. Missense mutations were the predominant variant type, with mutation frequencies of 8-22% across TLR1-TLR10 (**Supplementary Figure S1G**). As shown in **Supplementary Figure S1H**, Toll-like receptors have higher SNV frequencies in Uterine Corpus Endometrial Carcinoma (UCEC) and SKCM. Missense mutations also a key component in calculating TMB. So, we conducted a correlation analysis between TLRs expression and TMB in tumor. Overall, the correlation is weak among cancer types (**Supplementary Figures S2A-J**). In LUAD, TLR3 and TLR4 showed the highest mutation frequencies among TLR family members. We therefore assessed whether these alterations influence gene expression or clinical outcome. TLR3 and TLR4 mRNA levels did not differ significantly between mutant and wild-type LUAD (*P* > 0.05; **Supplementary Figures S2K, L**). In addition, stratified by TLR3 or TLR4 expression, the relationship between TMB and overall survival in LUAD was analyzed. The results showed that patients with low TMB in the TLR3 high expression group had a good prognosis, while patients with low TMB in the TLR3 low expression group had a poorer prognosis (**Supplementary Figures S2M-P**). Collectively, these pan-cancer data provide a global view of TLR alterations and served as a discovery screen to identify tumor types, including LUAD, in which TLR signaling might have prognostic and biological relevance.

### 3.2 The distribution and survival analysis of TLRscore

**Figure 1:**
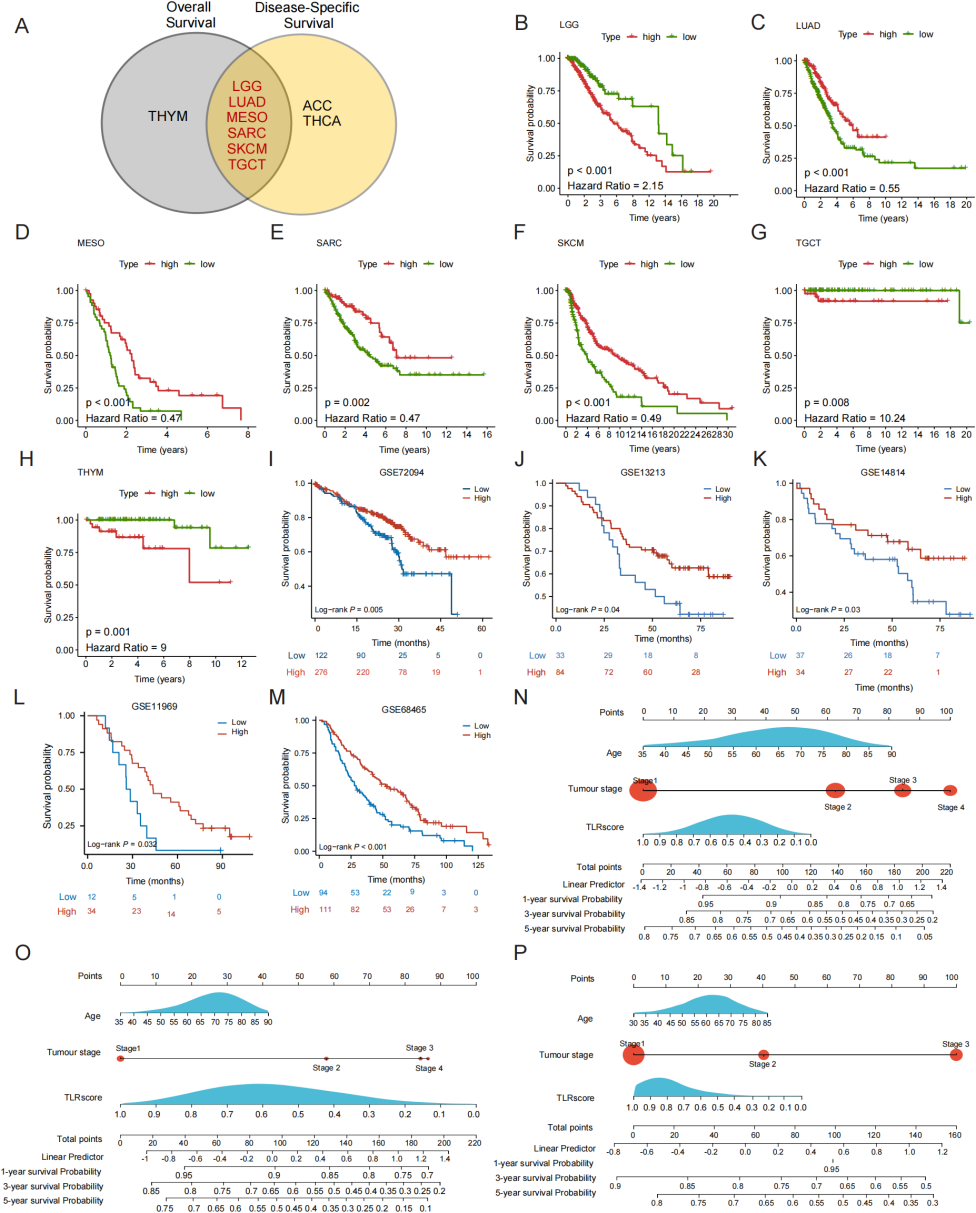
The distribution and prognostic value of TLRscore. **(A)** Univariate Cox regression analysis of the correlation between the TLRscore and OS and DSS. **(B-H)** Kaplan-Meier analysis of the association between TLRscore and overall survival in candidate cancer types. **(I-M)** Kaplan-Meier plot by TLRscore based on datasets of TCGA, GSE72094, GSE13213, GSE14814, GSE 11969, GSE68465. **(N-P)** Nomogram by multivariate cox regression analysis for predicting the proportion of OS.

To assess the expression levels of TLRs, we additionally conducted ssGSEA to determine the TLRscore across 32 different tumor types utilizing the TCGA cohort. As a technical validation of this scoring system, we first examined its relationship with the expression of each TLR gene. As expected from its construction, TLRscore showed strong positive correlations with all 10 TLRs, confirming that it faithfully reflects the overall TLR expression pattern (**Supplementary Figure S3A**).The results of univariate Cox regression analysis showed a significant correlation between TLR-score and OS and DSS in LGG, LUAD, MESO, SARC, SKCM, and TGCT (**Figure 1A**). As shown in **Supplementary Figure S3B**, a higher TLRscore was significantly associated with poorer OS in LGG and TGCT, whereas the opposite trend was observed in LUAD, Mesothelioma (MESO), SARC, and SKCM, where elevated TLRscore predicted a more favorable prognosis. To further minimize potential bias stemming from events unrelated to cancer, we also evaluated DSS and the results were similar to OS analysis (**Supplementary Figure S3C**). Subsequently, we conducted an analysis of Kaplan-Meier survival curves to compare groups with high and low TLR scores across 32 different types of cancer. Our data also showed that the high TLRscore predicted a better OS in LUAD, MESO, SARC and SKCM and worse OS in LGG, TGCT and THYM (**Figures 1B-H**). These results indicated that TLRscore were significantly associated with the prognosis of cancer patients. Subsequently, a comprehensive comparison across various cancers demonstrated significant variations in the TLRscore across the tumor types. The TLRscore reached its peak in Diffuse Large B-cell Lymphoma (DLBC) while recording its lowest value in Uterine Carcinosarcoma (UCS) (**Supplementary Figure S3D**). Next, we assessed the variations in TLRscore in tumor tissues compared to normal tissues, notable discrepancies were observed in the majority of cancers (**Supplementary Figure S3E**). Although the difference in TLRscore between normal and tumor tissue appears significantly higher in LUSC. However, compared to LUSC, LUAD consistently showed a favorable association between high TLRscore and improved survival and also exhibited marked differences in TLRscore between tumor and normal tissues. To test whether our findings depended on the specific scoring algorithm, we reconstructed the TLR signature using a z-score based averaging method applied to TLR1-TLR10 in LUAD. z-scores were highly correlated with the ssGSEA-based TLRscore (*r* = 1) (**Supplementary Figure S3F**). On this basis, we selected LUAD as the primary focus for subsequent detailed prognostic modeling and mechanistic studies.

Given the strong association between TLRscore and outcome observed in the pan-cancer screen, we next validated the prognostic value of TLRscore specifically in LUAD. The data from 6 cohorts (TCGA, GSE72094, GSE13213, GSE14814, GSE11969 and GSE68465) were analyzed by using Kaplan-Meier method. The results presented that the high-TLRscore predicted a better survival status, including early and recurrent LUAD (**Figures 1I-M**). Based on above analyses, we developed multivariate Cox models integrating age, tumor stage, and TLRscore for the TCGA, GSE72094, and GSE13213 cohorts, presenting them as prognostic nomograms (**Figures 1N-P**). By summing the points assigned to each variable, a “Total points” value is derived to predict 1-, 3-, and 5-year overall survival probabilities. Specifically, patients with early-stage disease and high TLRscores accumulate fewer points, indicating superior predicted survival, whereas advanced-stage patients with low TLRscores accumulate more points, corresponding to poorer outcomes. These nomograms convert the independent prognostic power of the TLRscore into a clinically applicable tool for LUAD risk stratification.

### 3.3 The relationship between carcinogenic driving factors and TLR score

**Figure 2:**
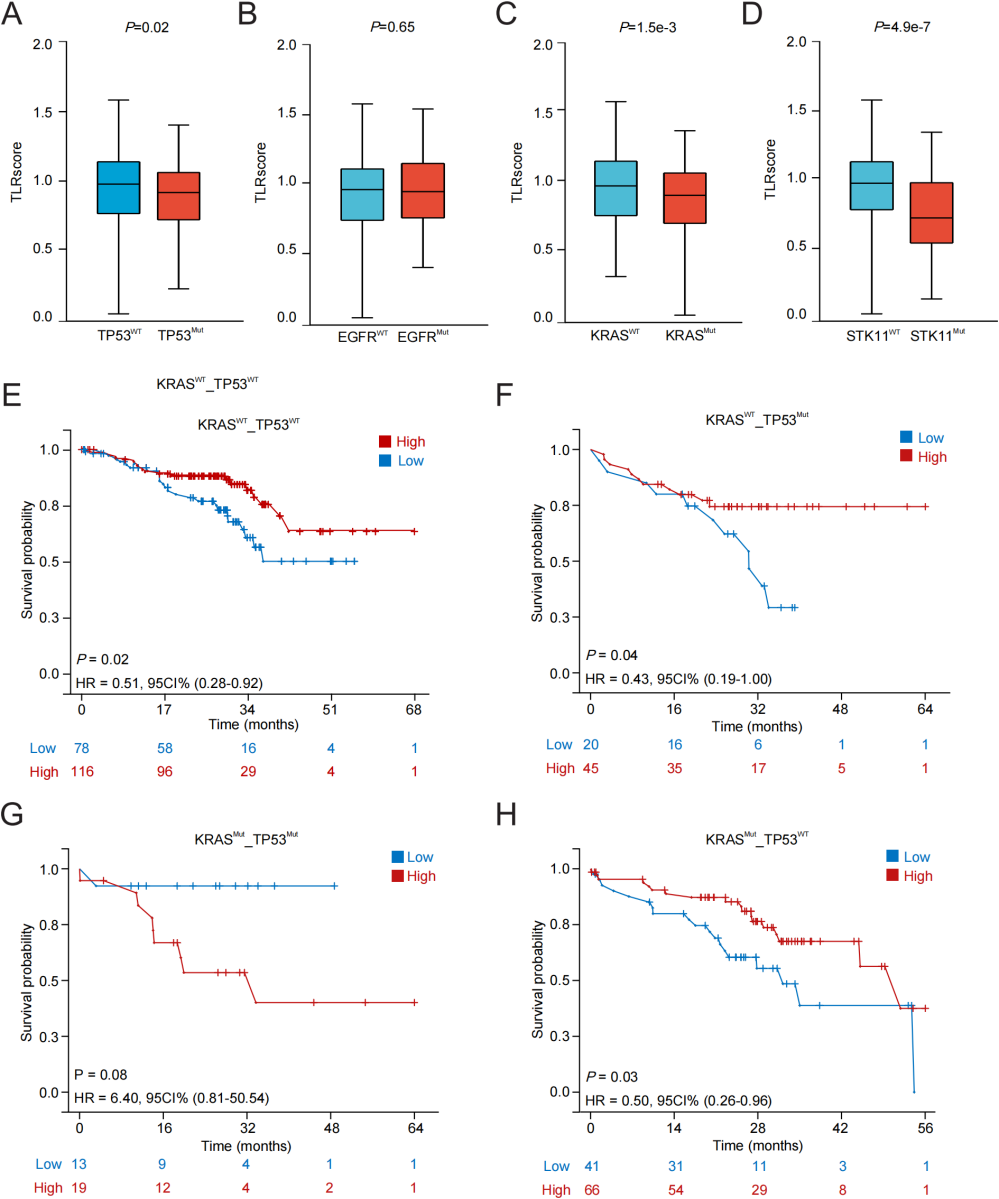
The relationship between carcinogenic driving factors and TLRscore. **(A-D)** The TLRscore and the mutation of TP53, KRAS, EGFR and STK11. **(E-H)** Prognostic analysis of patients stratified based on KRAS and TP53 mutation status.

The influences of TP53, KRAS, EGFR and STK11 mutations on TLRscore were also investigated based on GSE72094 dataset. The LUAD patients with wild type of TP53 (*P* = 0.02), KRAS (*P* = 1.5e-03) or STK11 (*P* = 4.9e-07) had higher TLRscore when compared with those with mutated one (**Figures 2A-D**). To further examine how major oncogenic drivers modulate the prognostic effect of TLRscore, we performed mutation-stratified survival analyses in the GSE72094 cohort. Because KRAS and TP53 frequently co-occur in LUAD, patients were first classified into four groups according to combined KRAS and TP53 status, and compared overall survival between high- and low-TLRscore subgroups (**Figures 2E-H**). High TLRscore was associated with significantly better survival in KRAS^WT^/TP53^WT^ (*HR* = 0.51, 95% *CI* = 0.28-0.92, *P* = 0.02), KRAS^WT^/TP53^Mut^ (*HR* = 0.43, 95% *CI* = 0.19-1.00, *P* = 0.04), and KRAS^Mut^/TP53^WT^ tumors (*HR* = 0.50, 95% *CI* = 0.26-0.96, *P* = 0.03). In contrast, in the KRAS^Mut^/TP5^3Mut^ subgroup, high TLRscore tended to correlate with poorer outcome (*HR* = 6.40, 95% *CI* = 0.81-50.54, *P* = 0.08), suggesting that the double-mutant setting may represent a distinct biological context.

### 3.4 Association of TLRscore with the immune microenvironment in LUAD

To discover the potential signaling pathways, hallmark pathway analysis was conducted. The results indicated that IFN-α/γ response, KRAS signaling up, TGF-β signaling, IL6-JAK-STAT3 signaling and immunoregulatory related pathways were enriched in TLRscore group, which indicated that TLRscore might importment in tumor‐induced immune and inflammatory process (**Supplementary Figure S4A**). To validate this finding, we performed the same analysis using GSE72094 and GSE13213 datasets and obtained similar results (**Supplementary Figures S4B, C**). Moreover, we investigate the relationships between TLRscore and immune-related genes. Specifically, chemokines, chemokine receptors, MHC genes, immunostimulatory factors and immunosuppressive factors were tightly correlated with TLRscore in LUAD (**Supplementary Figure S4D**).

**Figure 3:**
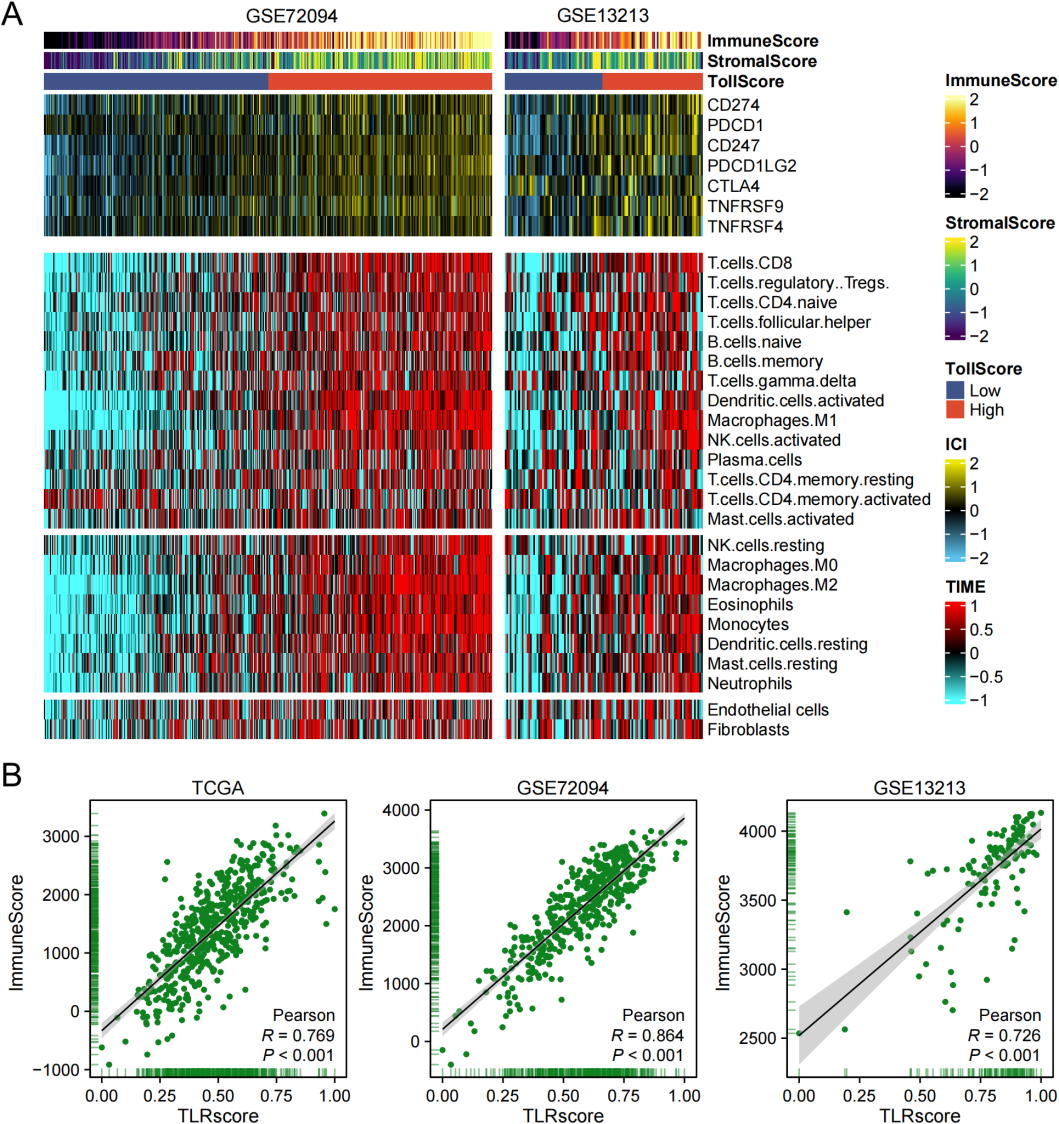
TLRscore-related immune infiltration analysis. **(A)** Immune checkpoint protein expression and tumor microenvironment by TLRscore. **(B)** The correlation between TLRscore with immune score in the GSE13213, GSE72094 and TCGA datasets.

Given that the pathway enrichment analysis indicated a strong association between TLRscore and inflammation as well as immune function, we conducted further exploration into the relationship between TLRscore and the infiltration of immune cells. Using GEO data sets (GSE72094, GSE13213), we found that the five immune cells (T cells, B cells, DCs, NK cells and Macrophages) were highly infiltrated in the high TLRscore group and the expression of immune checkpoints (CD274, PDCD1, CD247, PDCD1LG2, CTLA4, TNFRSF9, TNFRSF4) had high correlation with TLRscore (**Figure 3A**). According to ESTIMATE analysis, our findings indicated a positive correlation between TLRscore and ImmuneScore (*P* < 0.001, *r* = 0.726; *P* < 0.001, *r* = 0.864; *P* < 0.001, *r* = 0.769) in GSE13213, GSE72094 and TCGA-LUAD datasets (**Figure 3B**). In summary, the association of high TLRscore level with LUAD immune cell infiltration were further confirmed in our research.

### 3.5 TLRscore could predict the clinical benefit of immunotherapy

**Figure 4:**
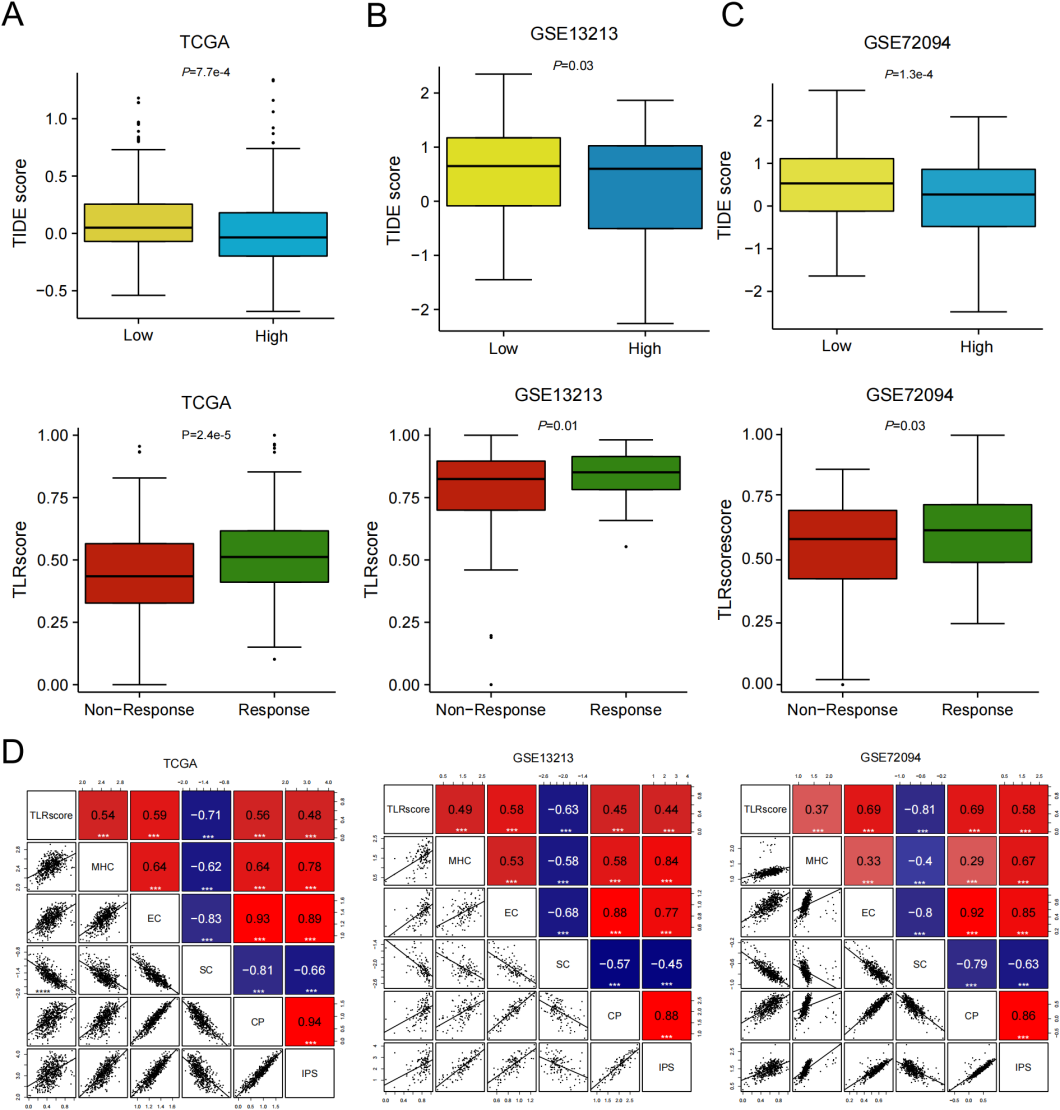
TLRscore for predicting the clinical benefit of immunotherapy. **(A-C)** Comparison of TIDE scores between the high and low TLRscore groups and TLRscore in the immunotherapeutic response versus non-response group in GSE13213, GSE72094 and TCGA datasets. **(D)** The correlation between TLRscore and IPS score in GSE13213, GSE72094 and TCGA datasets. ***P < 0.001.

CR, complete response; PD, progressive disease; PR, partial response; SD, stable disease.

ICI therapy is currently the most widely used and effective form of immunotherapy at present. To further evaluate the potential of TLRscore in predicting the benefits of immunotherapy, we used TIDE algorithm to predict the potential immune response and found that increased TLRscore was significantly correlated with the low TIDE scores, and LUAD patients with high TLRscore responded better to immunotherapy than those with low TLRscore (**Figure 4A**). When using validation sets (GSE13213 and GSE72094 cohorts), our data further verified the effect of TLRscore on the efficiency of immunotherapy (**Figures 4B, C**). IPS (MHC, EC, CP, and SC) serves as a more effective predictor for identifying the factors influencing immunogenicity and for characterizing the immunologic landscape within tumors. We found that the TLRscore was positively associated with the MHC, EC, CP score and negatively with SC score (**Figure 4D**), which suggested that patients with high TLRscore could be more responsive to ICI therapy.

**Figure 5:**
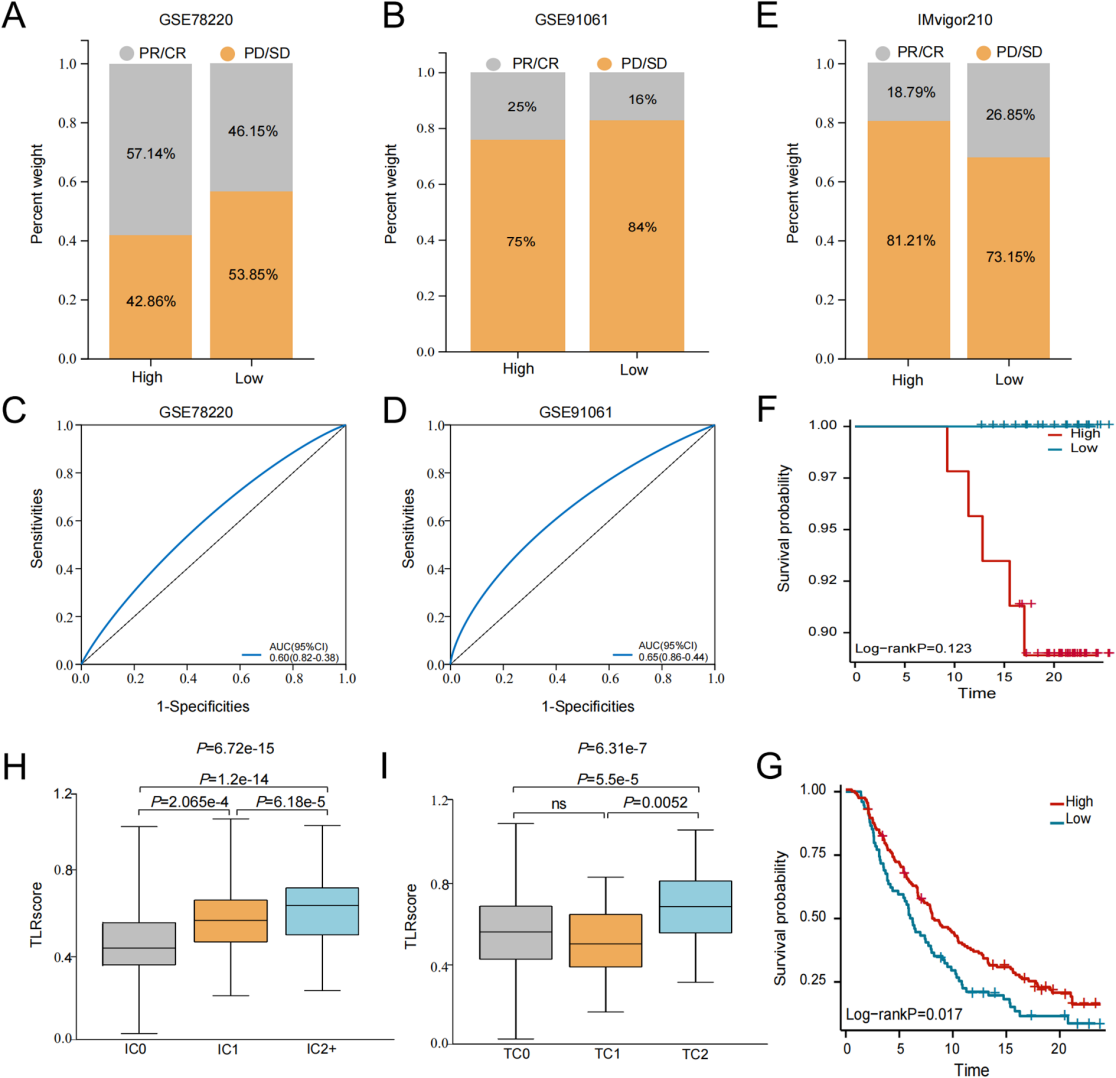
TLRscore and immunotherapy. **(A, B)** Correlation between the TLRscore and immunotherapy response in GSE78220 cohort and GSE91061 cohort. **(C, D)** ROC curves measuring the predictive value about objective response to ICB in GSE78220 and GSE91061 cohorts. **(E)** Correction between the TLRscore and immunotherapy response in IMvigor210 cohort. **(F, G)** Kaplan-Meier curve of overall survival by TLRscore in CR/PR **(F)** and SD/PD **(G)** groups of IMvigor210 cohort. **(H, I)** TLRscore difference in distinct PD-L1 scoring indicators. CR, complete response; PD, progressive disease; PR, partial response; SD, stable disease.

Furthermore, we also analyzed the percent of patients with partial response (PR)/complete response (CR) and progressive disease (PD)/stable disease (SD) across different TLRscore groups. As expected, in the anti-PD-1 cohort (GSE78220), patients with high TLRscore were more likely to benefit from immune checkpoint therapy, with a higher proportion PR/CR than PD/SD (57.14% vs 42.86%; **Figure 5A**). Interestingly, this association was not observed in the anti-CTLA4 melanoma cohort (GSE91061) and the anti-PD-L1 urothelial carcinoma cohort (IMvigor210) (**Figures 5B, E**). The sensitivity and specificity of the prediction models were assessed using AUC values greater than 0.600 (**Figures 5C, D**). In the IMvigor210 cohort, we performed a subgroup analysis based on therapeutic response. Among patients who responded to anti–PD-L1 therapy, those with a low TLRscore exhibited only a non-significant trend toward improved overall survival compared to the high-TLRscore group (*P* = 0.123; **Figure 5F**). This observation may be attributed to a notable imbalance in subgroup sizes, where the low TLRscore group contained considerably fewer patients than the high TLRscore group. In contrast, among non-responders, a high TLRscore was significantly associated with improved overall survival (*P* = 0.017; **Figure 5G**). In addition, based on the TIDE algorithm, the response to immunotherapy was analyzed for patients in the TCGA (**Supplementary Figure S5A**) and GSE72094 (**Supplementary Figure S5B**) datasets to further examine survival differences between high and low TLRscore groups according to immunotherapy response. The results indicated that patients with high TLRscores exhibited better survival regardless of whether they showed PD/SD or PR/CR. To relate TLRscore to PD-L1 expression, patients were further stratified into subgroups based on PD-L1 expression levels on immune cells (IC0, IC1, and IC2+) or tumor cells (TC0, TC1, and TC2+), as defined in the original cohort study (35). Notably, the highest TLRscore values were observed in the IC2+ (highest PD-L1-expressing immune cells) and TC2+ (highest PD-L1-expressing tumor cells) subgroups, with significant pairwise differences between the lowest and highest categories after Dunn’s *post-hoc* tests with FDR correction (*P* = 1.2e × 10^-14^ and 5.5e × 10^-5^, respectively; **Figures 5H, I**). Collectively, these findings offer initial support for the predictive significance of TLRscore in immunotherapy with ICIs.

### 3.6 Chemotherapy drug susceptibility prediction

**Figure 6:**
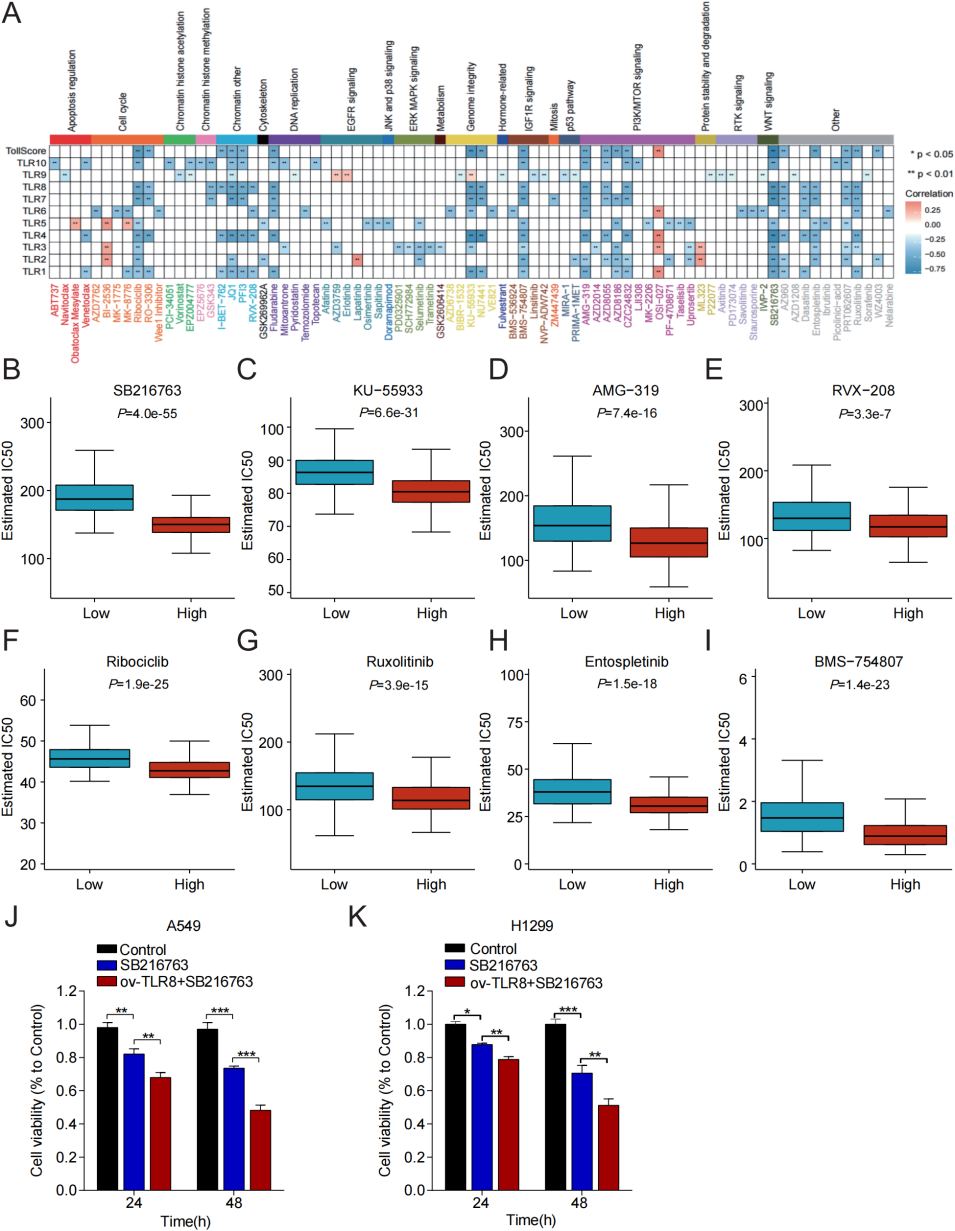
Sensitivity correlation analyses and prediction of potential drugs. **(A)** Correlation of TLRscore with the sensitivities of drugs in pan-cancer. **(B-I)** Sensitivity analysis of the 8 drugs with significant differences in the high and low TLRscore groups. **(J-K)** Proliferative ability of LUAD cells was measured by CCK-8 assay. *P < 0.05, **P < 0.01, ***P < 0.001.

To further enhance the clinical value of TLRs, we predicted the efficacy and signaling pathways of potential agents with the “pRRophetic” algorithm and compared the IC50 between the different TLRscore groups in LUAD (**Figure 6A**). The sensitivity of the drugs was inversely related to the IC50 value. For instance, we found that high TLRscore patients were more sensitive to SB216763 (GSK-3 inhibitor), KU-55933(ATM inhibitor), AMG-319 (PI3K inhibitor), RVX-208 (BET transcription inhibitor), Ribociclib (CDK inhibitor), Ruxolitinib (JAK inhibitor), BMS-754807 (IGF1R inhibitor) and Entospletinib (SYK inhibitor) than low-TLRscore patients (*P* < 0.05) (**Figures 6B-I**), which indicated that TLRscore might guide patients in obtaining more suitable drug therapy and served as a potential effective predictor of chemotherapy sensitivity prediction. Therefore, tWe treated lung adenocarcinoma cells with the GSK-3 inhibitor SB216763 for 24 and 48 hours, and assessed cell proliferation using the CCK-8 assay. The results demonstrated that SB216763 inhibited the proliferation of these cells, and that upregulation of TLR8 enhanced this inhibitory effect. (**Figures 6J, K**), supporting its potential as a therapeutic strategy for LUAD (**Figures 6J, K**), supporting its potential as a therapeutic strategy for LUAD.

### 3.7 Genetic regulation mechanism of TLR8 expression in LUAD

**Figure 7:**
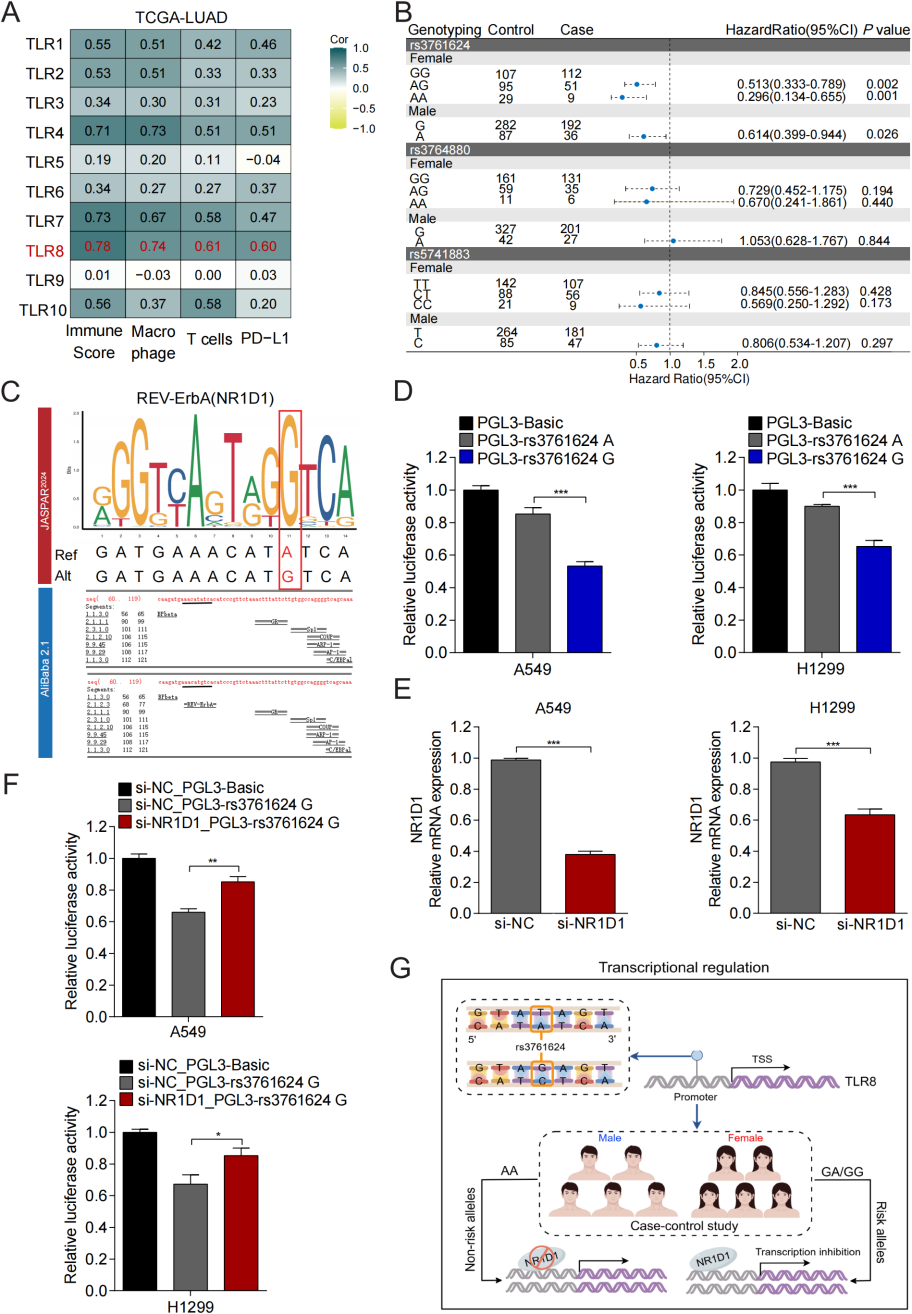
Genetic regulation mechanism of TLR8 expression in LUAD. **(A)** Correlation analysis between TLRs and immunescore, macrophage, T cells and PD-L1 expression. **(B)** The relationship between genetic variations in the TLR8 promoter region and the risk of developing lung adenocarcinoma. **(C)** Transcription factor prediction using JASPAR and AliBaba showed that rs3761624 was consistent with the binding sequence of NR1D1. **(D)** The effect of rs3761624 genetic variation on the activity of TLR8 gene promoter. **(E)** Knockdown efficiency of NR1D1. **(F)** The regulatory effect of transcription factor NR1D1 on TLR8 expression. **(G)** Mechanism diagram of TLR8 expression regulation. *P < 0.05, **P < 0.01, ***P < 0.001.

Our correlation analysis of TLRs with immune scores, macrophage presence, PD-L1 expression, and T cell activity revealed that TLR8 exhibited the strongest correlations among all TLRs (**Figure 7A**), this suggests that TLR8 may serve as a pivotal gene influencing the immune therapy response in patients with LUAD. Next, we explored whether TLR8 expression is regulated through epigenetic changes at the transcriptional level. Research has confirmed that Single nucleotide polymorphisms (SNPs) in the promoter region can significantly influence the binding affinity of transcription factors to DNA, thereby regulating gene expression levels. The genotype distribution of TLR8 SNP loci and their association with susceptibility to lung adenocarcinoma are illustrated in **Figure 7B**. Unconditional logistic regression analysis revealed that, in female patients, carriers of the AG or AA genotypes exhibited a significantly lower risk of developing lung adenocarcinoma compared to GG genotype carriers (AG: *OR* = 0.513, 95% *CI* = 0.333-0.789, *P* = 0.0023; AA: *OR* = 0.296, 95% *CI* = 0.134-0.655, *P* = 0.0017). Additionally, male patients who are A allele carriers demonstrated a reduced risk of lung adenocarcinoma compared to G allele carriers (*OR* = 0.614, 95% *CI* = 0.399-0.944, *P* = 0.026). However, no statistically significant difference (*P* > 0.05) was observed in the distribution of TLR8 rs3764880 and TLR8-AS1 rs5741883 genotypes between the case and control groups.

Given that rs3761624 is located in the promoter region of TLR8, the allele-specific activity of genetic variations in this region can influence the binding affinity of transcription factors (TFs). To further investigate the effect of rs3761624 genetic variations on transcription factor binding in the TLR8 promoter region, we utilized the JASPAR and Alibaba transcription factor prediction websites. Our research results indicate that the TLR8 promoter sequence carrying the rs3761624 G allele binds to NR1D1, whereas the sequence carrying the rs3761624 A allele does not (**Figure 7C**). Subsequently, we conducted dual luciferase reporter gene assays to assess the impact of the rs3761624 polymorphism on TLR8 transcriptional activity.

The wild-type luciferase reporter gene plasmid (pGL3-Rs3761624 A) or the mutant plasmid with the transcription factor NR1D1 binding site (pGL3-Rs3761624 G), encompassing the constructed TLR8 promoter region (-1443bp to -77bp), was co-transfected with the pRL-SV40 control plasmid into A549 and H1299 cells. The results demonstrated that, compared to the group transfected with pGL3-Rs3761624 A, the luciferase activity in the pGL3-Rs3761624 G group decreased was significantly reduced (*P* < 0.05). These findings indicate that transfection of the rs3761624 G allele is associated with significantly lower promoter activity, suggesting that this variant has a functional impact on TLR8 expression (**Figure 7D**). Transfecting NR1D1 specific siRNA into LUAD cell lines, qRT-PCR analysis showed that compared with the control group, the expression level of NR1D1 in the knockdown group decreased by 61.6% and 34.9% in A549 and H1299 cells, respectively (*P* < 0.01) (**Figure 7E**). Further transfection of the mutant plasmid (pGL3-rs3761624 G) into corresponding si-NR1D1 or si-NC cells revealed that compared with the si-NC group, the transcription activity of TLR8 gene was increased in the si-NR1D1 group (**Figure 7F**). These data demonstrate that the rs3761624-G allele enhances recruitment of the inhibitory transcription factor NR1D1 to the TLR8 promoter and suppresses TLR8 transcription, providing a mechanistic explanation for the reduced TLR8 expression and increased LUAD risk observed in G-allele carriers (**Figure 7G**).

### 3.8 Motolimod induces macrophage polarization and enhances anti-tumor immune response

**Figure 8:**
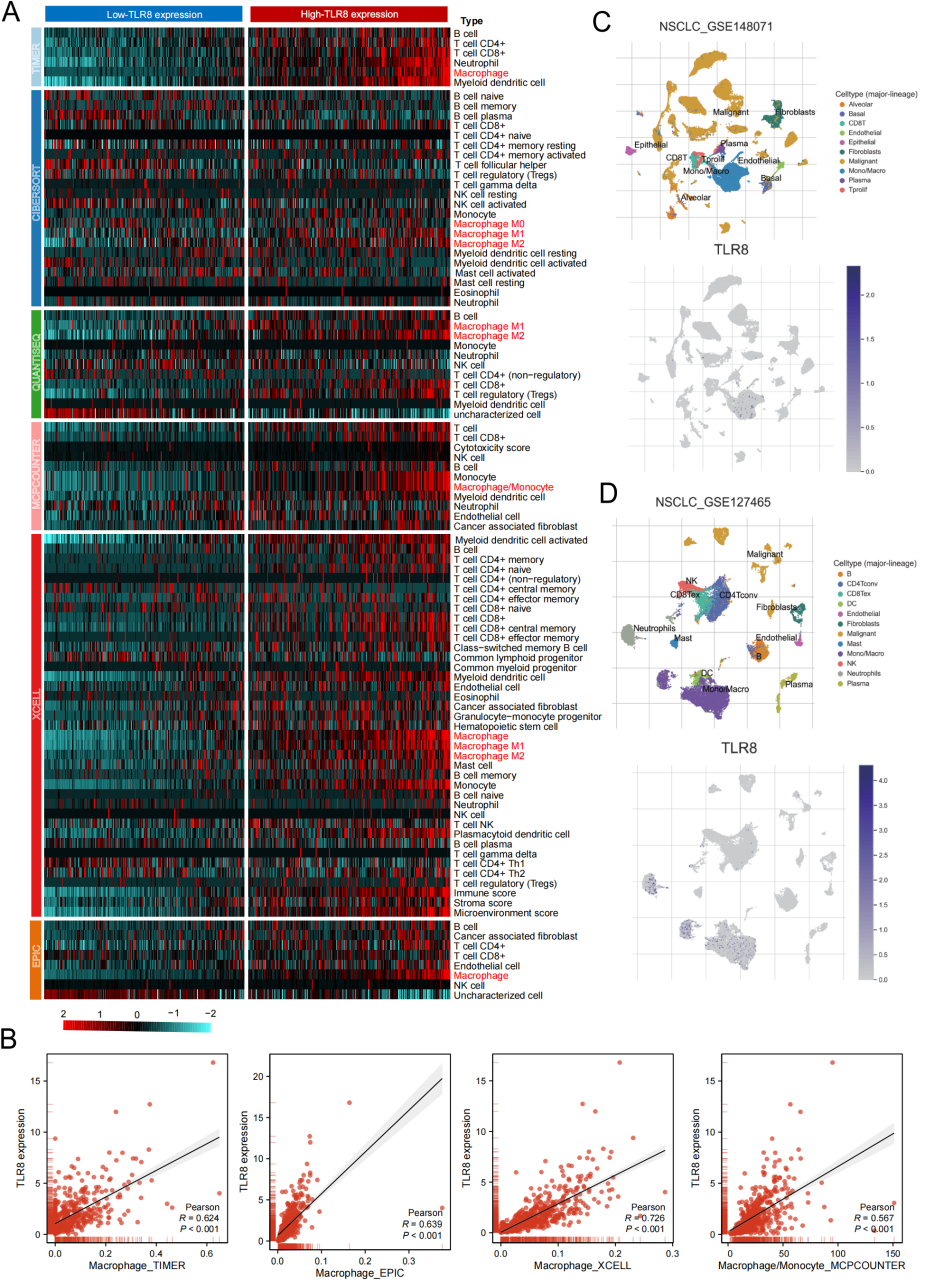
Differential Expression of TLR8 in Immune Cells. **(A)** The relationship between TLR8 expression and immune cell infiltration. **(B)** Correlation analysis between TLR8 expression and the level of macrophage infiltration. **(C, D)** Single-cell transcriptome analysis of TLR8 expression levels in immune cells.

Our germline genetic analyses indicate that the rs3761624 allele in the TLR8 promoter region significantly reduces TLR8 transcriptional activity, thereby contributing to inter-individual variability in TLR8 expression. Previous studies have shown that patients with lower endogenous TLR8 expression (such as those carrying risk alleles) may be precisely the group that needs to enhance the therapeutic effect of ICI by activating TLR8 to construct a favorable innate immune microenvironment (36, 37). To verify the feasibility of TLR8 as an immunotherapy target. We further investigated the relationship between TLR8 expression and immune cell infiltration using transcriptome data from TCGA lung adenocarcinoma. Utilizing algorithms such as TIMER, CIBERSORT, MCPCOUNTER, Xcell, EPC, and QUANTISEQ, we quantified the immune microenvironment of TCGA lung adenocarcinoma tissue samples and assessed their correlation with TLR8 expression. A pronounced immune infiltration pattern was observed in patients exhibiting high TLR8 expression (**Figure 8A**). Notably, macrophages demonstrated a stronger correlation with TLR8 expression compared to other immune cells, including T cells, B cells, and NK cells (**Figure 8B**). Single cell transcriptome analysis based on non-small cell lung cancer datasets (GSE148071 and GSE127465) showed that TLR8 is mainly expressed in monocytes/macrophages, which further validated our analysis (**Figures 8C, D**).

**Figure 9:**
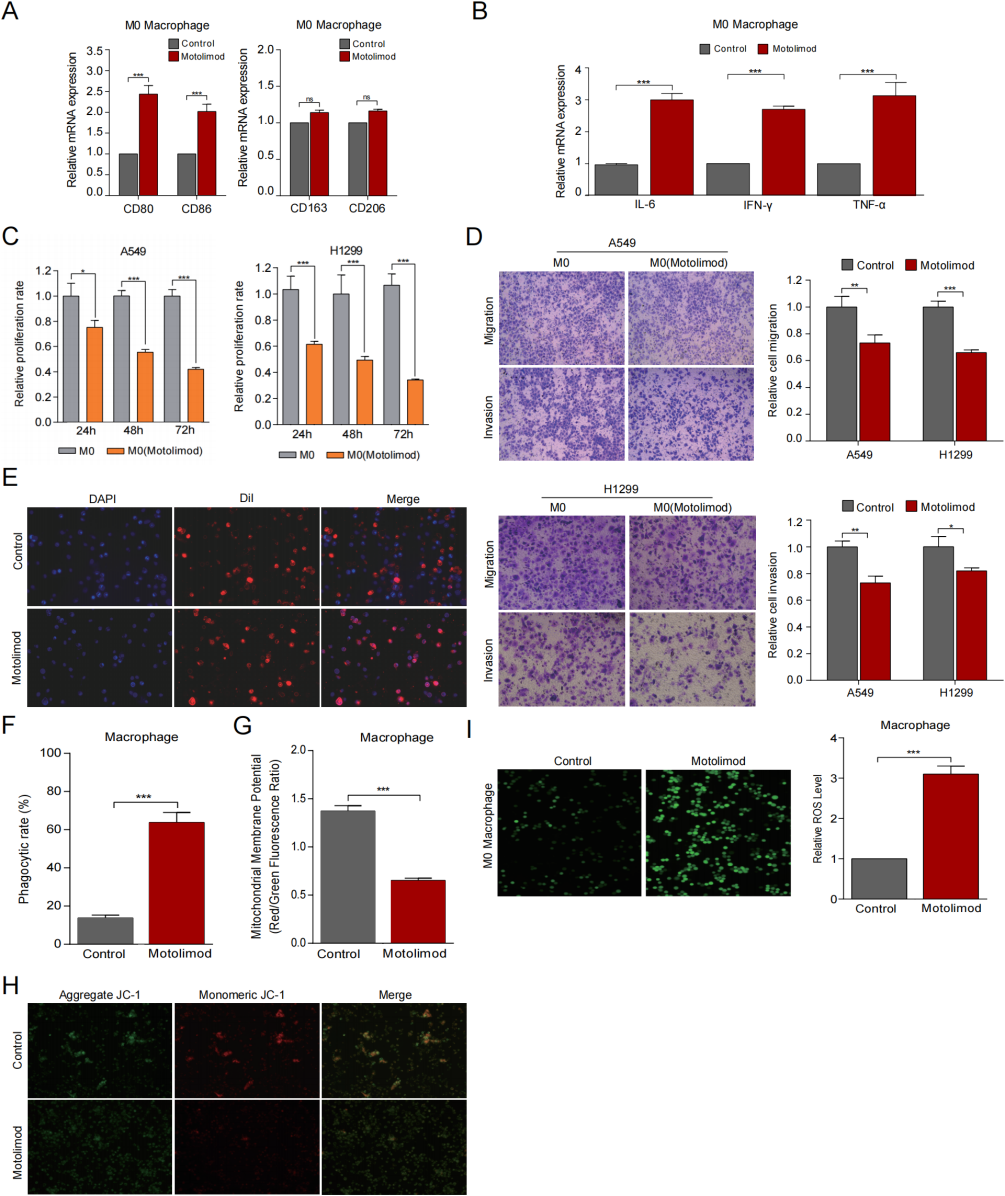
Motolimod induces M1 polarization of macrophages and enhances their antitumor activity via mitochondrial regulation. **(A)** Assessment of the polarization state in M0 macrophages exposed to Motolimod. **(B)** Measurement of pro-inflammatory cytokine mRNA expression levels in M0 macrophages post Motolimod treatment. **(C)** CCK-8 assay was employed to evaluate the proliferation of lung adenocarcinoma cells co-cultured indirectly with M0 macrophages that were treated or untreated with Motolimod. **(D)** Transwell assays assessed the migration and invasion capabilities of lung adenocarcinoma cells in indirect co-culture with M0 macrophages, with or without Motolimod treatment. **(E, F)** Evaluation of the phagocytic capacity of macrophages. **(G, H)** JC-1 staining utilized for the assessment of mitochondrial membrane potential. **(I)** DCFH-DA fluorescent probe was employed to measure ROS levels in macrophages. The data are expressed as mean ± SEM. *P < 0.05, **P < 0.01, ***P < 0.001.

Given the role of TLR8 in driving pro-inflammatory cytokine production and antitumor immune activation, and the possibility that low TLR8 expression may reduce patients’ responsiveness to immunotherapy, we next used the TLR8 agonist Motolimod to functionally stimulate macrophages and assess whether exogenous activation could enhance immune effector functions. To investigate the effect of TLR8 agonist (Motolimod) on M0 macrophage polarization, we measured the mRNA expression of M1 markers (CD80, CD86) and M2 markers (CD163, CD206) via qRT-PCR. As shown in **Figure 9A**, Motolimod treatment significantly increased CD80 and CD86 mRNA expression (*P* < 0.05) compared to the control group. For M2 markers, Motolimod had no significant effect on CD163 and CD206 expression. These results suggest that Motolimod may polarize M0 macrophages towards M1 macrophages. Next, we constructed an indirect co-culture system involving lung adenocarcinoma cells and M0 macrophages, utilizing conditioned medium derived from either Motolimod-treated or untreated M0 macrophages to culture the lung adenocarcinoma cells and evaluate their growth. Furthermore, compared with the control group, the use of Motolimod treatment resulted in a significant increase in cytokine expression (IL-6, IFN-γ, TNF-α) in macrophages cells (**Figure 9B**). Furthermore, compared with the control group, the morphological observations revealed that Motolimod treatment led to an increase in macrophage volume and the formation of plate-like pseudopodia, indicative of typical M1-type classical activation (**Supplementary Figure S6**). As illustrated in **Figure 9C**, the proliferation of lung adenocarcinoma cells in the Motolimod treatment group was significantly inhibited at 24, 48, and 72 hours compared to the M0 co-culture group alone. Meanwhile, we also observed that the migration and invasion capabilities of LUAD cells in the Motolimod treatment group were also suppressed (**Figure 9D**). These findings suggest that Motolimod may enhance the release of anti-tumor cytokines from polarized M1 macrophages, thereby triggering a more robust anti-tumor immune response in lung adenocarcinoma cells. Additionally, we established a direct co-culture system between lung adenocarcinoma cells and M0 macrophages to investigate the direct phagocytic effects of macrophages on lung adenocarcinoma cells. The results indicated that macrophages treated with Motolimod exhibited a stronger phagocytic ability towards A549 cells compared to the control group (**Figures 9E, F**). Previous studies have shown that the stable mitochondrial function of macrophages supports moderate ROS generation, which in turn drives M1 polarization and anti-tumor immune responses (38, 39). Therefore, we utilized the JC-1 fluorescent probe to assess changes in mitochondrial membrane potential in macrophages in response to TLR8 agonists. The results indicated (**Figures 9G, H**) that, compared to the control group, M0 macrophages exhibited an increase in the green fluorescence of the JC-1 monomer and a decrease in the red fluorescence of the JC-1 polymer upon treatment with the TLR8 agonist Motolimod. Additionally, merged images revealed that the green fluorescence of the JC-1 monomer predominated in M0 macrophages treated with Motolimod, suggesting a decrease in the mitochondrial membrane potential of these macrophages. Given the strong correlation between mitochondrial dysfunction and the enhancement of intracellular ROS production, we further investigated the levels of reactive oxygen species in macrophages using DCFH-DA probes. Our findings demonstrated that macrophages produce substantial amounts of ROS when exposed to TLR8 agonists, with the intensity of green fluorescence being directly proportional to the levels of reactive oxygen species within the cells (**Figure 9I**). The above results indicate that TLR8 may become a potential target for tumor immunotherapy, by regulating mitochondrial function or ROS levels, reshaping the anti-tumor phenotype of macrophages, and enhancing the efficacy of immunotherapy.

## 4 Discussion

Numerous studies have demonstrated that therapies targeting immune checkpoints can prolong anti-cancer responses by alleviating inhibitory signals within the immune system (40–42). However, due to the uncertainty of clinical response, there are still significant limitations in predicting the effectiveness of immunotherapy. Compared with single-omics approaches, integrative multi-omics analysis enables a comprehensive characterization of disease molecular landscapes across genomic alterations, epigenetic regulation, and transcriptional states, thereby facilitating the development of more robust and biologically interpretable prognostic models (43, 44). Therefore, based on the association between Toll like receptor expression and immune therapy responsiveness, we established the TLRscore as an integrative measure of TLR expression.

Notably, the prognostic effect of TLRscore was not uniform across tumor types. This bidirectional behavior is consistent with the context dependent biology of TLR signaling, which has been described as a “double-edged sword” in cancer. Our study demonstrates that high TLRscore was positively correlated with immune infiltration, activation of IFN-γ and JAK-STAT3 signaling, and enhanced antigen presentation, all of which are critical for effective anti-tumor immunity and likely account for the improved prognosis observed in these patients. Conversely, chronic inflammation induced by aberrant TLR signaling amplifies the IL-6/STAT3 pathway, driving MDSC expansion, immune checkpoint upregulation, and effector T cell suppression, collectively promoting immune escape (45). Moreover, we further noted that the prognostic impact of TLRscore depended on KRAS/TP53 status in LUAD. High TLRscore remained protective in most mutational backgrounds, whereas in the small KRAS^Mut^/TP53^Mut^ subgroup it tended to associate with poorer outcome. KRAS and TP53 co-mutations shows distinct biology and a highly inflamed immune profile compared with other co-mutation patterns (46). KRAS and TP53 co-mutation display higher tumor mutational burden, increased PD-L1 and immune-checkpoint expression and greater T-cell engagement (47). In KRAS/TP53 double-mutant LUAD, higher TLRscore might mark a particularly aggressive, TLR-high inflammatory niche that is detrimental in the absence of immune checkpoint blockade. Given the limited number of double-mutant cases in our dataset, this interpretation remains speculative, but it highlights that the clinical relevance of TLRscore is likely shaped by co-occurring driver mutations and treatment context. Therefore, the prognostic impact of TLRscore varies across different tumor types, depending on the tumor’s immune phenotype, genetic background, and tissue origin.

The TLRscore shows inconsistent predictive performance among different cancer types and immunotherapy cohorts, which may result from the interplay of multiple mechanisms. First, PD-1 inhibitors mainly block the binding of PD-1 on T cells to its ligands (PD-L1/PD-L2), thereby restoring effector T-cell activity (48). In contrast, PD-L1 inhibitors primarily target PD-L1 expressed on tumor cells or antigen-presenting cells, but do not completely block PD-L2-mediated inhibitory signaling (49), leading to fundamental mechanistic and biological differences between the two classes of immune checkpoint inhibitors (50). Second, distinct cancer types display marked TME heterogeneity in terms of immune cell infiltration, proportions of immunosuppressive cells (such as MDSCs and Tregs), expression of costimulatory molecules, and activation of inflammatory signaling pathways (51). Consequently, the predictive capacity of the TLRscore susceptible to modulation by immunosuppressive mechanisms within the tumor microenvironment. Finally, TLR signaling itself engages in complex crosstalk with immune checkpoint pathways. While TLR activation can promote antigen presentation and effector T-cell activation, excessive TLR signaling may induce PD-L1 expression, enhance IL-10 secretion, or activate immunosuppressive feedback mechanisms (52–54). These factors suggest that differences in immune checkpoint blockade mechanisms and the immunosuppressive pathways lead to the predictive value of the TLRscore across different cancer types and immunotherapy cohorts.

Our study indicate that a high TLRscore is significantly associated with improved overall survival in patients with lung adenocarcinoma, and also exhibited significant enrichment of CD8+ T cells, activated CD4+ T cells, and NK cells. It was also associated with the activation of both pro-inflammatory and immunosuppressive pathways, including KRAS and IL-6 signaling. A high TLRscore reflects an inflammation-driven immune-activated microenvironment characterized by the coexistence of immune stimulatory and inhibitory signals. the expression of immunosuppressive molecules is often driven by preceding immune activation events. IFN-γ signaling has been shown to upregulate immune checkpoint molecules such as PD-L1, CTLA-4, and LAG3 via enhanced cytotoxic activity (GZMB, PRF1), constituting a mechanism of “adaptive immune resistance” (55). Therefore, enhanced immune effector activity can induce compensatory upregulation of multiple immune checkpoint molecules, forming negative feedback loops that limit excessive immune responses. This mechanism accounts for the concurrent upregulation of immune stimulatory and inhibitory markers observed in our study, representing a dynamic equilibrium between immune activation and regulation within the tumor microenvironment.

ROS production has been recognized as a critical driver of M1 macrophage polarization and anti-tumor immunity (38, 39). This immunometabolic link expands the current understanding of how TLR8 reshapes the tumor immune landscape and provides new rationale for targeting innate immune pathways in LUAD. This is also consistent with broader evidence that redox-regulatory pathways shape inflammatory responses, such as the MALAT1/miR-140-5p/Nrf2 axis described by Qin et al. in ischemia–reperfusion injury, where modulation of oxidative stress confers tissue protection (56). Although studied in a different context, these findings support the concept that targeting mitochondrial function and ROS–Nrf2 signaling can be leveraged to tune myeloid-cell behavior, in our case toward TLR8-driven antitumor immunity in LUAD.

Toll-like receptor (TLR)-mediated signaling has been recognized as an important upstream mechanism that induces reactive oxygen species (ROS) production and amplifies immune responses. Previous studies have shown that TLR7 agonists can induce mitochondrial ROS (mtROS) production in monocyte-derived dendritic cells, thereby promoting downstream IL-12–dependent antitumor immune responses (57). In addition, TLR activation can drive mitochondrial translocation toward phagosomes through the TRAF6/ECSIT pathway, leading to increased mtROS generation and enhanced immune activation (58). Simultaneously, ROS can participate in macrophage metabolic reprogramming by regulating key transcription factors such as NRF2 and HIF-1α, thereby further influencing macrophage polarization (56, 59, 60).Collectively, these findings suggest that ROS may serve as a critical bridge linking TLR signaling to metabolic and phenotypic reprogramming in macrophages. Mechanistically, we demonstrated that TLR8 activation perturbs mitochondrial homeostasis and induces moderate ROS accumulation in macrophages, which is critical for sustaining pro-inflammatory polarization and anti-tumor immune activity. Previous studies have demonstrated that TLR8 agonists can enhance the secretion of pro-inflammatory cytokines such as TNF-α and IL-12, and induce an M1-like phenotype in tumor-associated macrophages or monocytes (20, 61, 62). Moreover, preclinical studies have shown that TLR8 agonists can effectively reprogram anti-inflammatory, pro-tumoral M2 macrophages into pro-inflammatory, antitumor M1 phenotypes (63). Although the precise causal sequence among mitochondrial dysfunction, ROS generation, and TLR8-mediated macrophage polarization remains to be fully elucidated, integrating previous studies with our experimental results allows us to tentatively propose that TLR8 activation may modulate mitochondrial homeostasis and ROS levels, thereby contributing to macrophage pro-inflammatory polarization and the induction of antitumor immune responses. Future studies will aim to delineate the complete mechanistic in detail.

Given that the TLRscore correlates strongly with immune activation and antitumor immune responses in our study, modulation of TLR signaling may have important therapeutic implications. Various studies suggest that SNPs within the promoter region may affect mRNA expression by altering the interactions between transcription factors and the promoter (64, 65). We show that genetic and epigenetic variation in the TLR8 promoter (rs3761624) directly influences transcription factor binding and gene expression. Together with our Motolimod experiments showing that pharmacologic TLR8 activation reprograms macrophages toward an M1-like, antitumor phenotype, the rs3761624 findings suggest that individuals with genetically reduced TLR8 expression (rs3761624-G carriers) may have impaired endogenous TLR8 signaling yet could be particularly amenable to therapeutic rescue with TLR8 agonists, a hypothesis that will require future genotype-stratified clinical evaluation. This observation aligns with prior reports linking germline variants in innate immune receptors to cancer susceptibility and therapeutic response (66, 67). Although our study centered on TLR8, TLR8 signaling likely operates within a broader network of TLR pathways. Previous studies have shown that extracellular vesicles derived from colorectal cancer can increase macrophage secretion of TNF - α and IL-6 in a m6A modification dependent manner through TLR8, thereby promoting cancer cell proliferation (68). Similarly, stimulation of lung cancer cell lines with TLR7 or TLR8 agonists leads to NF-κB signaling activation, upregulation of anti apoptotic protein Bcl-2 expression, increased tumor cell survival, and chemoresistance (69). Although this is inconsistent with the results of this article, studies have shown that chronic, low-level TLR8 stimulation may induce tolerance or promote tumor inflammation, while acute, intense, especially drug delivery stimulation may break tolerance and trigger effective anti-tumor immunity (70). In addition, lung cancer with high mutation load may produce more new antigens and abnormal nucleic acid substances as endogenous ligands for TLR8, which are captured by antigen-presenting cells to initiate immunity (71). Combinatorial activation of distinct TLRs can elicit synergistic antitumor responses: co-stimulation of TLR7/8 (Telratolimod) and TLR9 (CpG ODN) enhances dendritic cell maturation and CD8^+^ T-cell activation, leading to superior tumor control compared with single agents (72). The higher efficacy of TLR3 (poly(I:C)) combined with TLR7/8 (R848) versus single treatments to polarize macrophages toward M1-like antitumor effectors *in vitro (*73). Likewise, intratumoral injection of R848 and poly(I:C) synergistically promoted antitumor immune responses by reprogramming macrophage polarization and activating DCs in lung cancer (74). In addition, the combination of R848 with sorafenib enhances antitumor effects by reprogramming the tumor immune microenvironment and facilitating vascular normalization in hepatocellular carcinoma (75). These findings suggest that TLR8-directed strategies in LUAD could be further optimized by rationally combining TLR8 agonists with other TLRs agonists or chemotherapeutic drugs, while carefully considering potential synergistic as well as antagonistic crosstalk.

Nonetheless, this study has certain limitations. First, the genetic association analysis for rs3761624 was restricted to a Han Chinese LUAD cohort. Further studies are required to validate the clinical relevance of this variant in diverse ethnic populations. Second, although TLR8 activation in macrophages was accompanied by mitochondrial depolarization, increased ROS, and M1-like polarization, these data are associative and do not establish a strict causal sequence among these events; future studies using ROS scavengers and mitochondrial modulators will be needed to confirm whether mitochondrial and ROS changes are required mediators of TLR8-driven macrophage reprogramming. Third, regarding the TLRscore, TLR8 expression, and macrophage-related signatures rely primarily on public transcriptomic datasets and *in vitro* models, lacking validation in an independent clinical cohort. The translational relevance of the TLRscore and TLR8 as biomarkers should be regarding as preliminary; future investigations are necessary to validate these findings at the protein and tissue levels in clinical LUAD specimens. We will address these limitations in our subsequent research.

## 5 Conclusion

In summary, our study positions TLR8 as a pivotal regulator of LUAD immune microenvironment remodeling and introduces TLRscore as a novel biomarker for patient prognosis and therapeutic responsiveness. Future clinical trials incorporating TLR8 agonists, such as Motolimod, in combination with ICB or chemotherapy, may provide synergistic benefit and expand the scope of precision immunotherapy in LUAD.

## Data Availability

The original contributions presented in the study are included in the article/**Supplementary Material**. Further inquiries can be directed to the corresponding author/s.

## Supplementary material

Supplementary Figure 1 **(A)** The heatmap showing the differentially expressed fold change of TLRs in each kind of cance. **(B)** A survival analysis of TLRs across 33 tumor types from TCGA was conducted using univariate Cox regression and Kaplan-Meier methods. **(C)** A heatmap illustrating the differential methylation status of TLRs across 23 different cancers. **(D)** The correlation between the expression of TLRs and the methylation of their promoters is illustrated. **(E)** TLRs somatic copy number variant analysis. **(F)** The correlation analysis between CNA and TLRs expression. **(G)** Depicted in an oncoplot is the mutational profile of TLRs, alongside a typology of SNVs. **(H)** Analysis of mutations in TLRs. Numbers represent the number of samples that have the corresponding mutated gene for a given cancer.

Supplementary Figure 2 **(A-J)** Correlation analysis between TLR1-TLR10 expression and TMB in pan-cancer. **(K-L)** The relationship between TLR3 and TLR4 mutations and expression. **(M-N)** Stratified by TLR3 or TLR4 expression, the relationship between TMB and overall survival in LUAD was analyzed.

Supplementary Figure 3 **(A)** Heatmap showing the relationship between Toll like receptor gene expression and TLRscore. **(B-C)** Univariate Cox regression analysis of the correlation between the TLRscore and OS **(B)** and DSS **(C)**. **(D)** The TLRscore in different cancer types based TCGA. (E) The TLRscore comparison between cancer and normal tissues. **(F)** Consistency analysis of TLR-score and Z-score based on ssGSEA.

Supplementary Figure 4 **(A)** Signal pathway enrichment analysis for differentially expressed genes between high- and low-TLRscore. NES is the normalized enrichment score in the GSEA algorithm. **(B, C)** KEGG analysis in GEO dataset. **(D)** Correlation between TLRscore and immunomodulators, including chemokine, receptor, MHC, immunoinhibitor, and immunostimulator.

Supplementary Figure 5 **(A)** Kaplan-Meier curve of overall survival by TLRscore in CR/PR and SD/PD patients with TCGA-LUAD. **(B)** Kaplan-Meier curve of overall survival by TLRscore in CR/PR and SD/PD patients with GSE72094.
