# Supplementary figures and images for: Multi-omics analysis identifies TLRscore for prognostic prediction and highlights TLR8 in macrophage-mediated antitumor immunity of lung adenocarcinoma
Source: Front Immunol. 2026 Feb 10;17:1711401. doi: 10.3389/fimmu.2026.1711401 (PMC12929545; doi:10.3389/fimmu.2026.1711401)

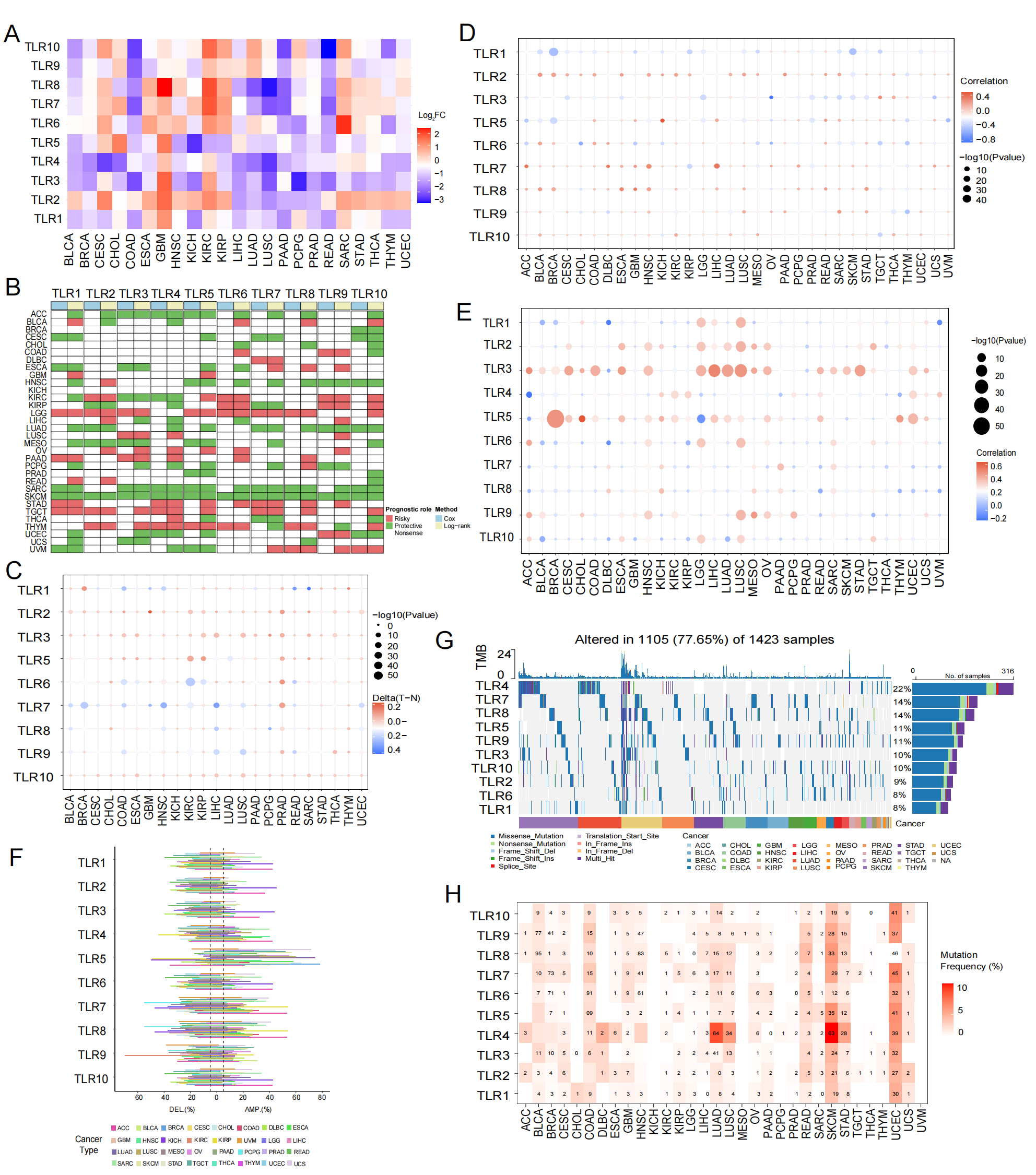

Supplement: Supplementary Figure 1 — (A) The heatmap showing the differentially expressed fold change of TLRs in each kind of cance. (B) A survival analysis of TLRs across 33 tumor types from TCGA was conducted using univariate Cox regression and Kaplan-Meier methods. (C) A heatmap illustrating the differential methylation status of TLRs across 23 different cancers. (D) The correlation between the expression of TLRs and the methylation of their promoters is illustrated. (E) TLRs somatic copy number variant analysis. (F) The correlation analysis between CNA and TLRs expression. (G) Depicted in an oncoplot is the mutational profile of TLRs, alongside a typology of SNVs. (H) Analysis of mutations in TLRs. Numbers represent the number of samples that have the corresponding mutated gene for a given cancer. [file Image1.tif]

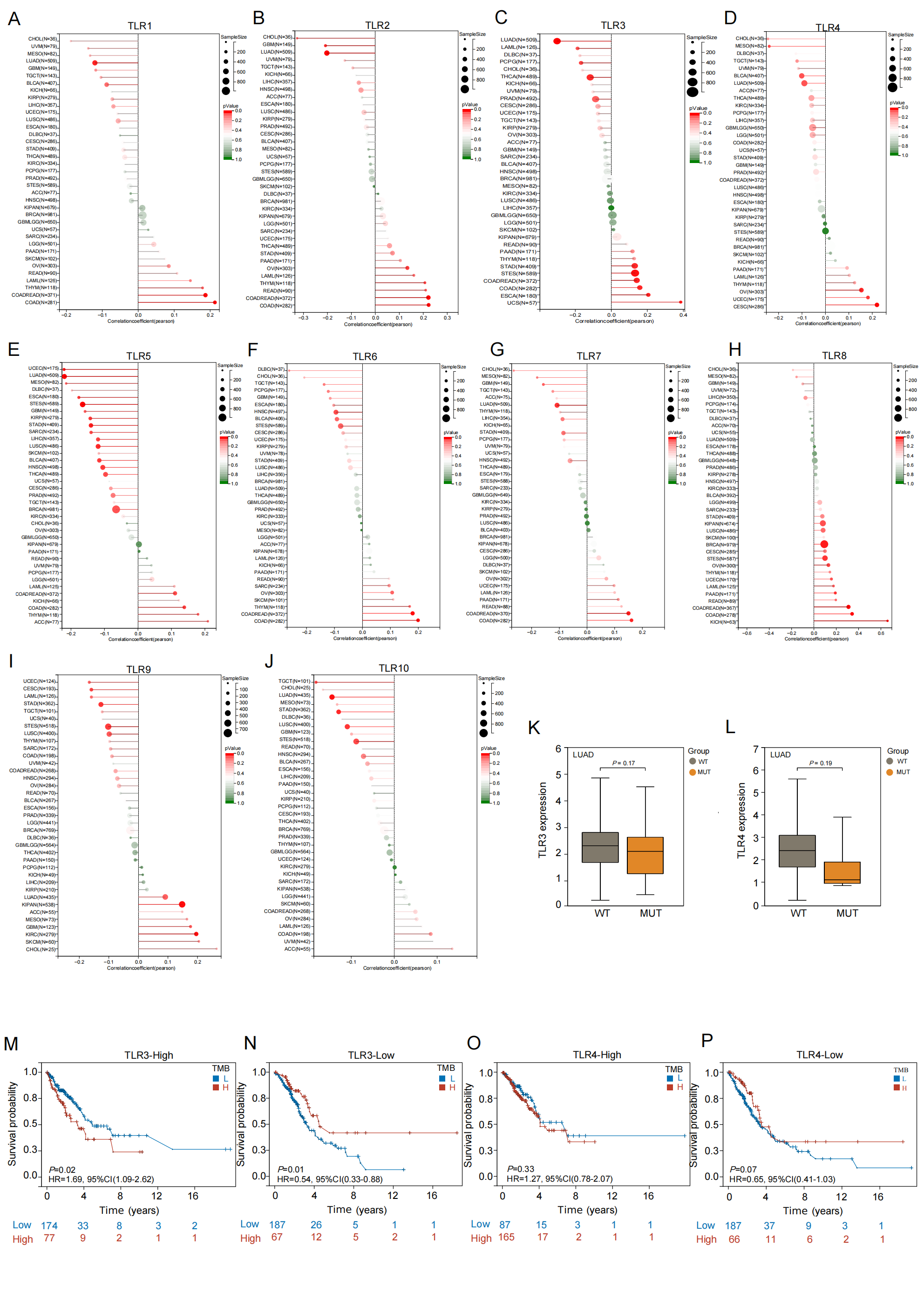

Supplement: Supplementary Figure 2 — (A-J) Correlation analysis between TLR1-TLR10 expression and TMB in pan-cancer. (K-L) The relationship between TLR3 and TLR4 mutations and expression. (M-N) Stratified by TLR3 or TLR4 expression, the relationship between TMB and overall survival in LUAD was analyzed. [file Image2.tif]

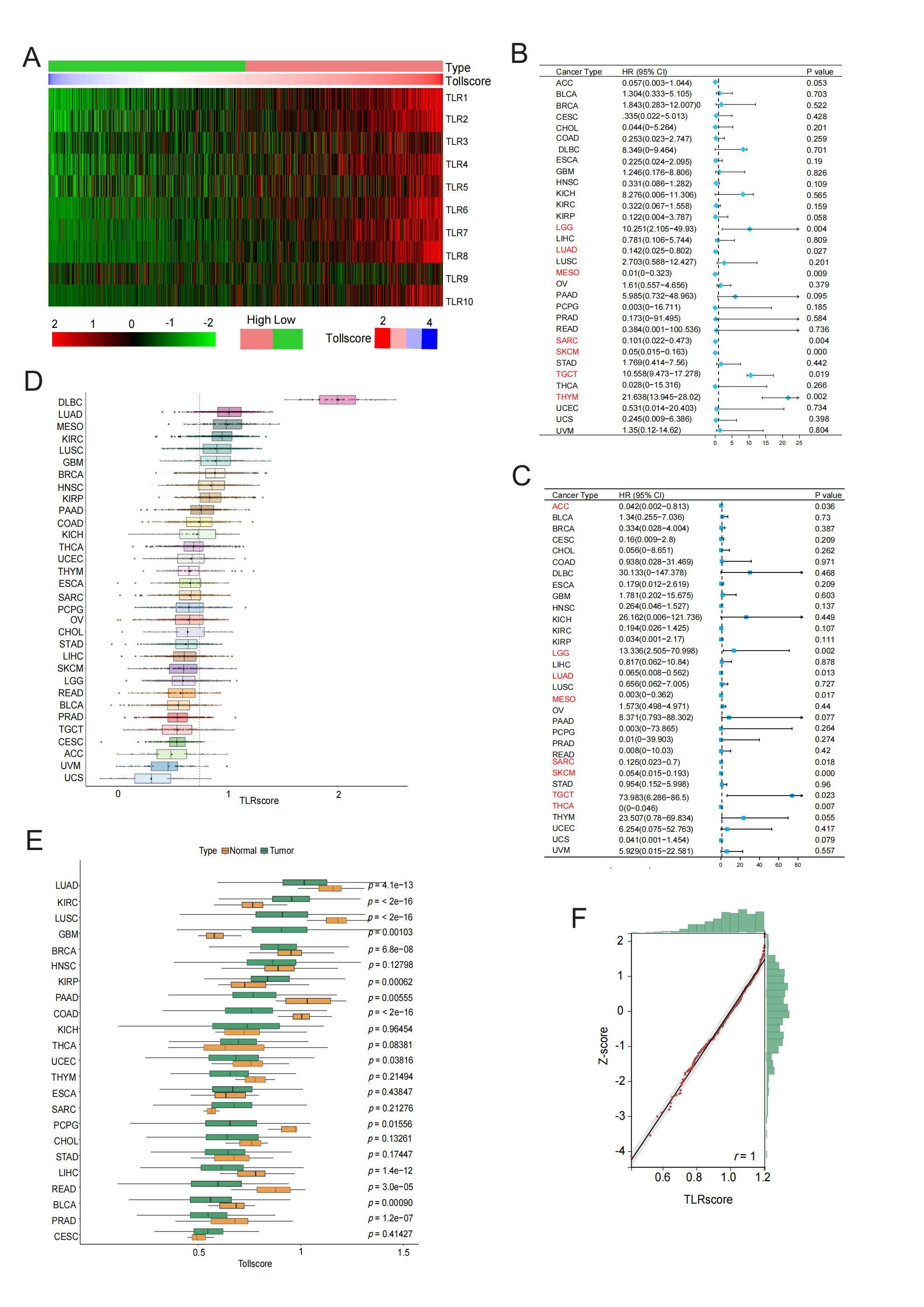

Supplement: Supplementary Figure 3 — (A) Heatmap showing the relationship between Toll like receptor gene expression and TLRscore. (B-C) Univariate Cox regression analysis of the correlation between the TLRscore and OS (B) and DSS (C). (D) The TLRscore in different cancer types based TCGA. (E) The TLRscore comparison between cancer and normal tissues. (F) Consistency analysis of TLR-score and Z-score based on ssGSEA. [file Image3.tif]

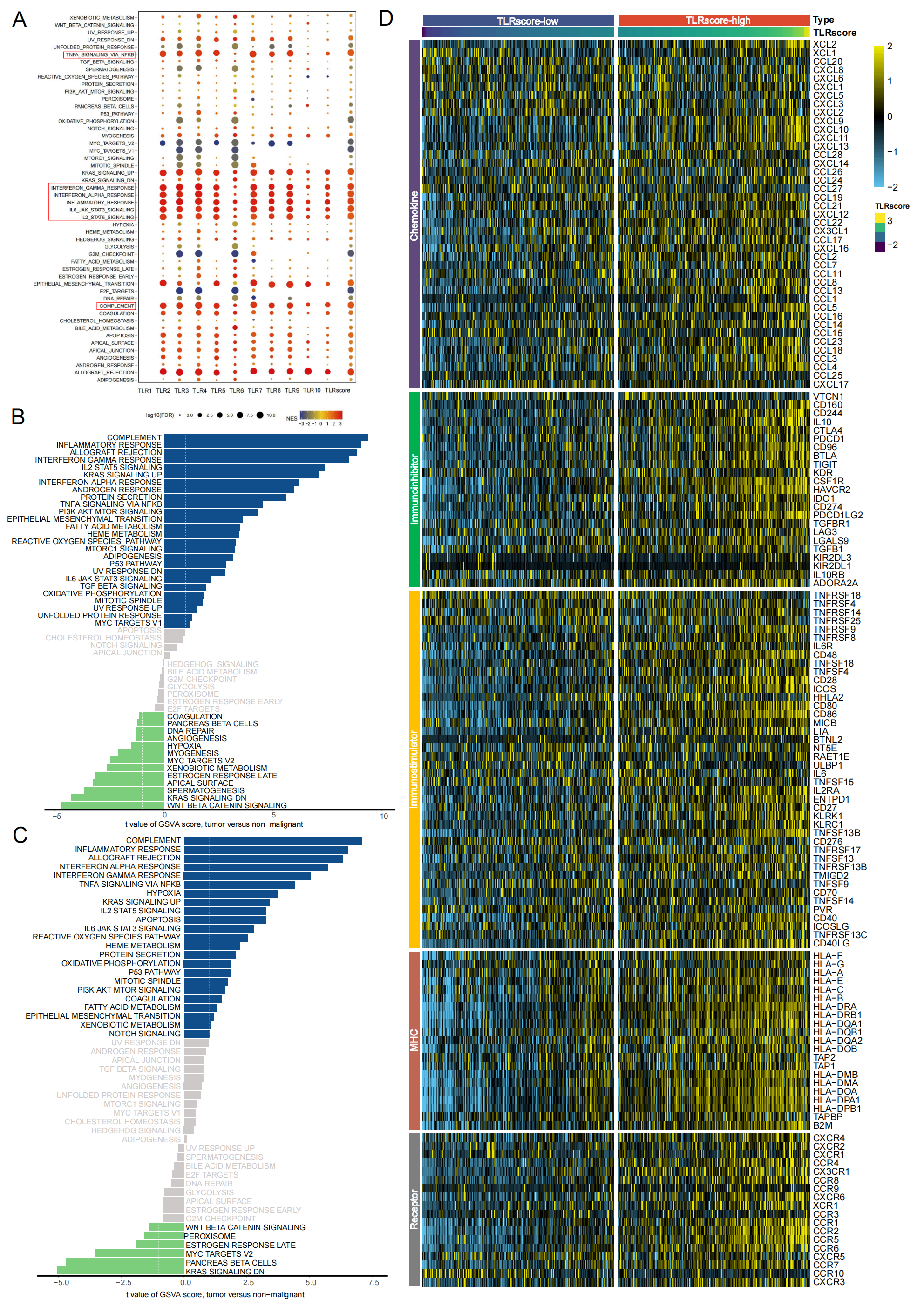

Supplement: Supplementary Figure 4 — (A) Signal pathway enrichment analysis for differentially expressed genes between high- and low-TLRscore. NES is the normalized enrichment score in the GSEA algorithm. (B, C) KEGG analysis in GEO dataset. (D) Correlation between TLRscore and immunomodulators, including chemokine, receptor, MHC, immunoinhibitor, and immunostimulator. [file Image4.tif]

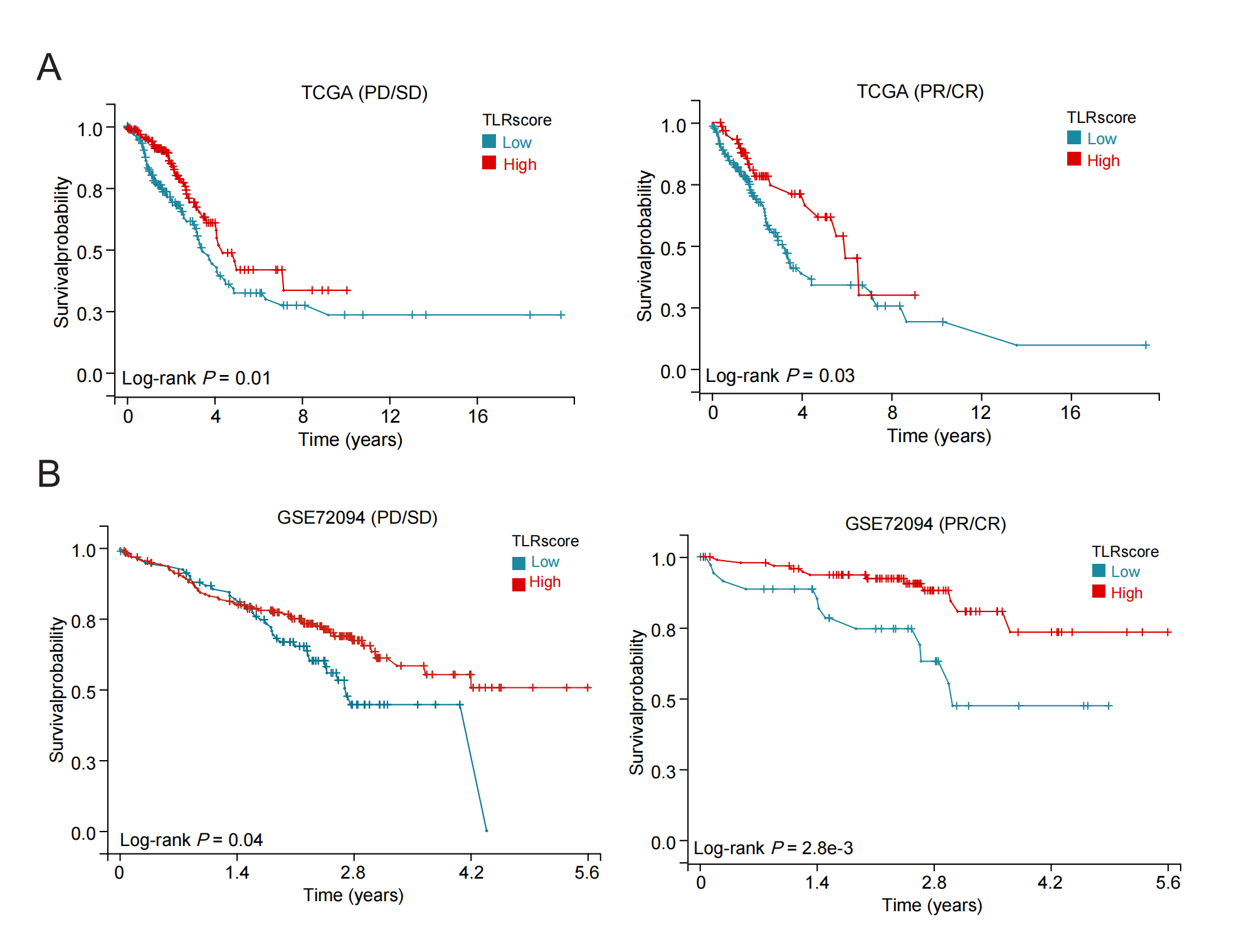

Supplement: Supplementary Figure 5 — (A) Kaplan-Meier curve of overall survival by TLRscore in CR/PR and SD/PD patients with TCGA-LUAD. (B) Kaplan-Meier curve of overall survival by TLRscore in CR/PR and SD/PD patients with GSE72094. [file Image5.tif]

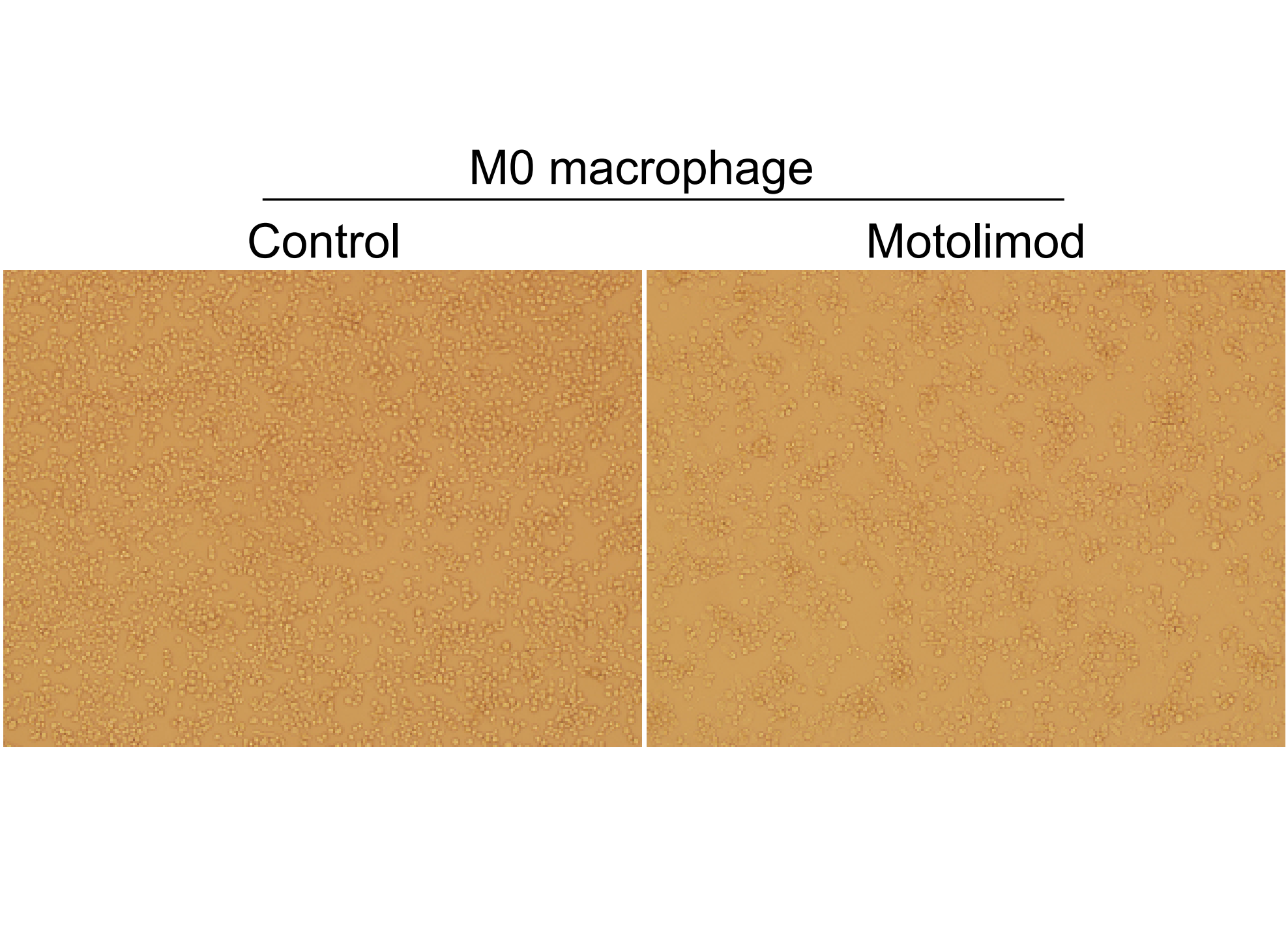

Supplement: Supplementary file 6 [file Image6.tif]
