# Supplementary material for: Multi-omics analysis identifies TLRscore for prognostic prediction and highlights TLR8 in macrophage-mediated antitumor immunity of lung adenocarcinoma
Source: Front Immunol. 2026 Feb 10;17:1711401. doi: 10.3389/fimmu.2026.1711401 (PMC12929545; doi:10.3389/fimmu.2026.1711401)
Supplement: Supplementary file 7 [file Table1.docx]

**Supplementary Table**

**Supplementary Table 1. Basic characteristics of the research object**

| Variable | Control group（N=600） | |  | Case group（N=400） | | *P* valuea |
| --- | --- | --- | --- | --- | --- | --- |
|  | Number | （%） |  | Number | （%） |  |
| Sex |  |  |  |  |  | 0.155 |
| Man | 369 | 61.5 |  | 228 | 57.0 |  |
| Female | 231 | 38.5 |  | 172 | 43.0 |  |
| Age |  |  |  |  |  | 0.497 |
| ≤60 | 350 | 58.3 |  | 242 | 60.5 |  |
| >60 | 250 | 41.7 |  | 158 | 39.5 |  |
| Smoking Status |  |  |  |  |  | 0.410 |
| Non-smoking | 408 | 68.0 |  | 262 | 65.5 |  |
| Smoking | 192 | 32.0 |  | 138 | 34.5 |  |
| Accumulated smoking volume |  |  |  |  |  | 0.709 |
| ≤30 | 118 | 19.7 |  | 82 | 20.5 |  |
| >30 | 74 | 12.3 |  | 56 | 14.0 |  |
